# Equipped for success: genomes and metabolomes of the European *Amanita muscaria* are conserved in its novel South African range

**DOI:** 10.1111/nph.71064

**Published:** 2026-03-12

**Authors:** Grant R. Nickles, Cecelia K. Stokes, Deborah L. Narh, Kira M. T. Lynn, Savannah R. Fuqua, Corbin Bryan, Brooke M. Allen, Christopher P. Bivins, Jin Woo Bok, J. Stephen Brewer, Sikelela T. Buthelezi, Jahiya P. R. M. Clark, Kerri L. Coon, Lauren R. Corby, Martin P. A. Coetzee, Claudette Dewing, Tuan A. Duong, Mathew A. Harris, Nancy P. Keller, Katlego Kopotsa, Frances A. Lane, Holly L. Nichols, Alandie Nieuwoudt, Martin A. Nuñez, Miguel E. Medina Munoz, Sung Chul Park, Nam Q. Pham, Kaetlyn T. Ryan, Myriam Solís, Rytas Vilgalys, Jasmine Marea Wallace, Yen‐Wen Wang, Brenda D. Wingfield, Michael J. Wingfield, Travis K. Worley, Taylor A. Zallek, Mostafa Zamanian, Jason D. Hoeksema, Milton T. Drott, Anne Pringle

**Affiliations:** ^1^ Department of Medical Microbiology and Immunology University of Wisconsin‐Madison Madison WI 53706 USA; ^2^ Department of Botany University of Wisconsin‐Madison Madison WI 53706 USA; ^3^ USDA Forest Service Northern Research Station, Center for Forest Mycology Research Madison WI 53726 USA; ^4^ Department of Biochemistry, Genetics and Microbiology, Forestry and Agricultural Biotechnology Institute (FABI) University of Pretoria Private Bag X20 Pretoria South Africa; ^5^ Department of Biology Stanford University Stanford CA 94305 USA; ^6^ Department of Ecology and Evolutionary Biology University of Arizona Tucson AZ 85712 USA; ^7^ Department of Biology University of Mississippi University MS 38677 USA; ^8^ University of California‐Merced Merced CA 95343 USA; ^9^ Forestry and Agricultural Biotechnology Institute (FABI) University of Pretoria Pretoria South Africa; ^10^ Ecology and Evolutionary Biology Department University of California Santa Cruz Santa Cruz CA 95064 USA; ^11^ Department of Bacteriology University of Wisconsin‐Madison Madison WI 53706 USA; ^12^ Jackson State University Jackson MS 39217 USA; ^13^ Department of Plant and Soil Sciences University of Pretoria Private Bag X20 Pretoria 0002 South Africa; ^14^ Department of Plant Pathology University of Wisconsin‐Madison Madison WI 53706 USA; ^15^ Faculty of Applied Science Eduvos 22 Umgazi Rd, Ashlea Gardens Pretoria 0081 South Africa; ^16^ Microbiology Doctoral Training Program University of Wisconsin‐Madison Madison WI 53706 USA; ^17^ Department of Biology and Biochemistry University of Houston Houston TX 77204‐5001 USA; ^18^ Department of Pathobiological Sciences University of Wisconsin‐Madison Madison WI 53706 USA; ^19^ Biology Department Duke University Box 90338 Durham NC 27708 USA; ^20^ Mississippi Valley State University Itta Bena MS 38941 USA; ^21^ Department of Biostatistics Yale University New Haven CT 06520 USA; ^22^ Department of Plant Pathology University of Minnesota Minneapolis MN 55455 USA; ^23^ Cereal Disease Laboratory, Agricultural Research Service USDA Saint Paul MN 55108 USA

**Keywords:** bioactivity, biosynthetic gene clusters, fly agaric, Global Natural Product Social Molecular Networking analysis, invasive ectomycorrhizal fungi, invasive mushrooms, secondary or specialized metabolites or metabolism, South Africa

## Abstract

- Plants and soils have been moved around the world for centuries, but invasive mushrooms receive scant attention. The *Amanita muscaria* species complex was introduced to South Africa in the context of forestry, but its origins, ecology and recent evolution are unstudied.
- We sequenced the genomes of 24 Northern and Southern Hemisphere *A. muscaria*, built phylogenies and reconstructed its South African history. We identified the biosynthetic gene clusters (BGCs) encoding specialized metabolites (SMs). We subsequently extracted mushrooms' metabolites and used mass spectrometry data to group SMs into unique molecular families (MFs). We tested metabolites for bioactivity against diverse microbes and animals.
- We identify Europe as the origin of South African *A. muscaria*. A highly conserved group of BGCs is found in nearly all European and African genomes, and only 13 of 273 MFs are unique to South Africa. Metabolites extracted from all mushrooms kill nematodes, while microbes and flies appear unaffected.
- The nearly global distribution of the fly agaric results from multiple introductions of a single European clade to the Southern Hemisphere. Despite its long history in South Africa, the fungus has not lost any of its BGCs, suggesting a conservation of function(s) across multiple continents.

## Introduction

Humans have moved plants and soils among continents for centuries, resulting in the co‐introduction of fungal pathogens and symbionts (Anagnostakis, 1987; Dickie *et al*., 2010; Cleary *et al*., 2016). Fungi mediate basic ecological processes (Treseder & Lennon, 2015), and when introduced fungi become invasive, they can dramatically alter local ecosystems (Anagnostakis, 1987; Desprez‐Loustau *et al*., 2007; Rossman, 2009; Boyd *et al*., 2013). We follow Policelli & Nuñez (2025) and define an invasive fungus as a non‐native species able to ‘disperse, survive, and reproduce in multiple locations across a range of habitats’. Interest in invasive nonpathogenic fungi is growing (Pringle *et al*., 2009; Dickie *et al*., 2010; Vargas *et al*., 2019; Milani *et al*., 2022), but most research continues to focus on the biology and impacts of introduced and invasive pathogens. As mutualist and decomposer fungi also spread across the globe (Vellinga *et al*., 2009; Berch *et al*., 2017; Vargas *et al*., 2019; Pildain *et al*., 2021; Veerabahu *et al*., 2025), discovering the mechanisms enabling their successful invasions and documenting their impacts on native ecosystems emerge as key research priorities.

Among the most intensively studied nonpathogenic fungal invasions are co‐invasions involving ectomycorrhizal (ECM) fungi and plant species of *Pinus* (family Pinaceae). Pines have been introduced across the Southern Hemisphere for commercial forestry, reforestation and afforestation. These non‐native trees now cover more than 6 million hectares in countries, including South Africa, Argentina, Brazil, Chile and New Zealand (Rouget *et al*., 2001; Simberloff *et al*., 2010; Dickie *et al*., 2014; Brancatelli *et al*., 2020). Species like *Pinus contorta*, *P. elliottii*, *P. patula*, and *P. radiata* have spread beyond forestry plantations and into natural forest and grassland biomes, including into biodiversity hotspots, for example, South Africa's fynbos (Richardson & Higgins, 1998; van Wilgen & Richardson, 2012). Invading pines can reduce water availability, suppress native plant and fungal species, and disrupt ecosystem services(Le Maitre *et al*., 2002; Dickie *et al*., 2014; Nuñez *et al*., 2017; Brewer *et al*., 2018; Sapsford *et al*., 2022).

Pines are obligately associated with ECM fungi, a symbiosis enabling the trees to obtain essential nutrients and water (Smith & Read, 2008; Nuñez *et al*., 2009; Dickie *et al*., 2010). Historically, plantings of pines in the Southern Hemisphere failed until soils were imported to supply their symbionts (Mikola, 1970), and introduced ECM fungi have subsequently facilitated invasions of pine trees (Hayward *et al*., 2015; Policelli *et al*., 2019, 2023). Despite the critical role of ECM fungi in pine invasions and the potential for ECM fungi to invade and harm native forests on their own (Dickie *et al*., 2017), the ecological and evolutionary dynamics of invading ECM fungi remain poorly understood, perhaps because invading mutualists are perceived as unequivocally beneficial (but see Schwartz *et al*., 2006). In fact, ECM fungi may function across a spectrum of mutualism and parasitism (Karst *et al*., 2008). Efforts to elucidate the ecology of ECM fungi are further exacerbated by their ephemeral and often cryptic biology (Wang *et al*., 2023), leaving us with incomplete, often inaccurate historical records of species' native ranges (Pringle & Vellinga, 2006). Strategies combining historical research with modern ‘omics methods offer a novel approach to identify the drivers of ECM fungal invasions.

Invasion biology often invokes interactions among species as a mechanism driving spread, and fungal interactions are often hypothesized to be mediated by specialized metabolites (SMs; Drott *et al*., 2017; Keller, 2019; Tannous *et al*., 2023). Although ECM fungi produce a plethora of SMs (Schüffler, 2018; Walton, 2018; Obermaier & Müller, 2020; Mudbhari *et al*., 2024) and must routinely interact with pathogens, predators and competitors, interactions between introduced ECM fungi and local organisms are rarely considered. However, the toxins of at least one invasive ECM fungus, *Amanita phalloides*, appear to be evolving dynamically in its invasive range (Drott *et al*., 2023). This finding and the ubiquity of fungal SMs raise questions about the translation of the ‘enemy release’ and ‘novel weapons’ hypotheses, often invoked to explain the success of non‐native plants and animals, to invasive fungi (Blossey & Nötzold, 1995; Torchin *et al*., 2003; Callaway & Ridenour, 2004; Liu & Stiling, 2006). The ‘enemy release’ hypothesis proposes introduced species succeed by escaping natural enemies, while the ‘novel weapons’ hypothesis suggests they spread by producing compounds against which native species have no resistance (Gillett, 1962; Callaway & Ridenour, 2004).

The ecological roles and evolution of SMs remain poorly understood, even in iconic ECM species like *Amanita muscaria* *sensu lato* (s.l.; Geml *et al*., 2006), the famous red‐and‐white‐spotted ‘fly agaric’ known for its psychoactive properties (Camazine, 1983; Michelot & Melendez‐Howell, 2003) and for the folklore suggesting it can repel or kill flies (Lumpert & Kreft, 2016). *A. muscaria* produces ibotenic acid and muscimol using a single biosynthetic pathway (Størmer *et al*., 2004; Su *et al*., 2023), and muscimol is a potent GABA_A_ receptor agonist (Johnston, 2014), a feature which contributes to its distinctive biological effects. Native to nearly all temperate and boreal biomes of the Northern Hemisphere, the *A. muscaria* species complex includes multiple cryptic lineages associated with diverse plants, including pines. While some of these cryptic species are now formally recognized (e.g. *Amanita persicina*; Tulloss *et al*., 2015), the true number of cryptic species within the *A. muscaria* species complex remains unknown. For simplicity, we refer to all as‐yet unnamed lineages as *A. muscaria*. Introduced to South America, Australia and Africa, *A. muscaria* forms novel ECM partnerships with native trees in Columbia (*Quercus humboldtii*; (Vargas *et al*., 2019), Chile (*Nothofagus*) (Márquez Parraguez, 2024); and Australia (*Nothofagus* and *Allocasuarina*) (Fuhrer, 1992; Dunk *et al*., 2012; Lebel *et al*., 2024)). Relatively little is known about its introductions across Africa (Vellinga *et al*., 2009), but its South African history is particularly well documented (Box 1), offering a rare opportunity to study the impact of *A. muscaria*'s introduction on the fungus's evolution and production of SMs.

We sequenced the genomes of 24 *A. muscaria* s.l. mushrooms (9 South African, 11 European, 2 Australian and 2 Californian) and 1 outgroup (*Amanita pantherina*) to address three hypotheses: First, South African *A. muscaria* would be most closely related to a European lineage of the fungus. Our hypothesis is based on the history of *A. muscaria* in South Africa (Box 1). Second, a release from antagonistic interactions found in Europe but absent in South Africa would relax selection on SMs, enabling the fungus to invest more energy into growth and reproduction and potentially causing the loss of SM genes or gene function. Third, adaptation in the invasive range would result in shifts in metabolic profiles and impact the bioactivity of extracts against laboratory model bacteria, fungi, nematodes, mosquitoes and flies.

Box 1 **The history of *Amanita muscaria* in South Africa**

**Fig. 1:**
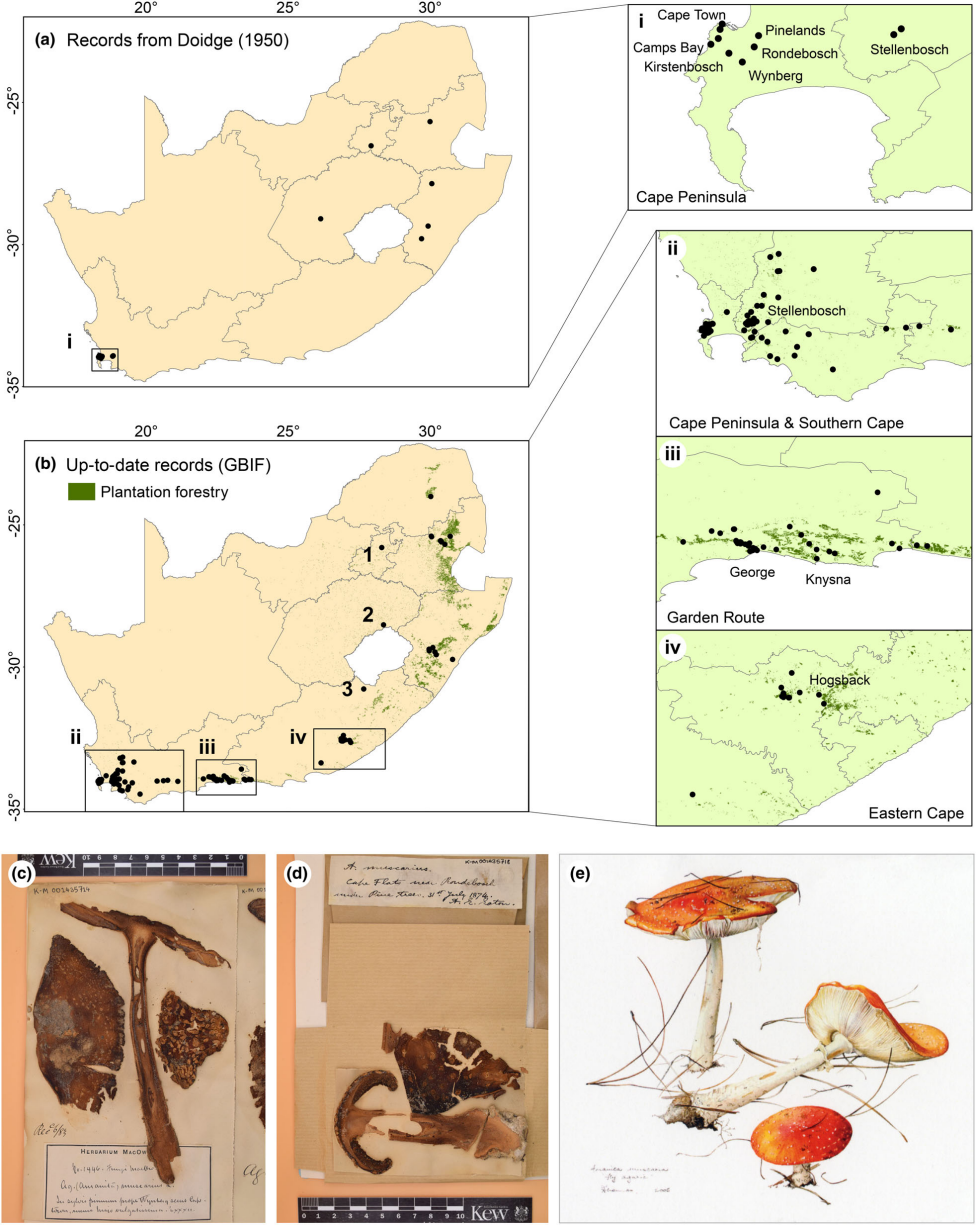
Records of *Amanita muscaria* in South Africa. Occurrence data illustrate the spread of *A. muscaria* in South Africa over the last century. Panel (a) plots historical records up until 1945 as compiled by Doidge (1950). Insert (i) is an enlargement of the Cape region. Panel (b) plots contemporary records as of 24 July 2023, also illustrating plantation forestry land use. Inserts are enlargements of (ii) Cape Town and the southern Cape, (iii) the Garden Route and (iv) the Eastern Cape. Numbered occurrences (1–3) are not associated with plantation forestry and instead record *A. muscaria* associated with pine trees in urban areas as in (1) Pretoria or (2) Clarens, or in rural areas as in (3), where pines are often used as windbreaks or to adorn the edges of farm roads. Panels (c) and (d) are images of *A. muscaria* specimens from Wynberg and Rondebosch in Cape Town collected in 1883 and 1874, respectively. Both records provide valuable information about associations with plants, for example ‘under Pine trees’ in panel (d). Panel (e) is an image from a card bought at Kirstenbosch Botanical Gardens in Cape Town in 2023, reflecting widespread awareness of the fungus in South Africa (image copyright Vicki Thomas (Aristea Publications) and used with her kind permission).

Doidge (1950) lists a number of early collections of the fungus; by 1874, *A. muscaria* was growing with pines in and around Cape Town (Fig. 1). By 1945, it was growing in what are now named as the provinces of Mpumulanga to the north and Free State in the east. She identifies *A. muscaria* as growing with ‘shrubs’, pine trees and ‘near living roots of *Quercus* sp.’ (Doidge, 1950 p. 553).

The history of *A. muscaria* is linked to the history of Europeans on the continent. Early European colonists did not find the timber species native to South Africa suitable for their uses, and they introduced fast‐growing pines. Any plant moved to South Africa by European colonists would have been accompanied by fungi in its tissues, and plants may also have been moved as seedlings growing in soil, enabling the introduction of soil fungi. Doidge (1950) succinctly describes the dynamic of plants arriving to South Africa with Europeans:

‘The history of plant introduction into South Africa begins in 1651, with the arrival of Johan van Riebeeck to establish a settlement at the Cape; van Riebeeck was accompanied by a gardener… To provide timber, sacks of acorns were sent from Holland and kernels of stone and cluster pines from Italy; these were planted in quantity and flourished amazingly… the majority being species of Pinus, Eucalypts and poplars…’ Doidge (1950, pp. 15–16)

Mikola (1970) goes on to describe the subsequent deliberate export of soils from South Africa to enable plantation forestry elsewhere, including in Zimbabwe and Kenya (Mikola, 1970; Zimbabwe discussed as Rhodesia). For example, three tons of soil from the Transvaal were imported to establish pine plantations in Eswatini (formerly Swaziland), and apparently *A. muscaria* was moved as well, ‘The commonest mushrooms in Usutu Forest of Swaziland [now Eswatini] in March 1967 were… *Laccaria laccata*, *Amanita muscaria*, *Boletus edulis*, *Rhizopogon roseolus*’. (Mikola, 1970).

The fungus is acknowledged as a European introduction in the most recent scientific literature on the *Amanita* of South Africa (Reid & Eicker, 1991) and in a current field guide (Goldman & Gryzenhout, 2019). While *A. muscaria* may have been introduced with European ‘stone and cluster pines’ (Doidge, 1950), in modern South African forestry plantations, it associates with pines from other continents, for example Mexican *Pinus patula* and Californian *P. radiata*.

## Materials and Methods

### Historical records of South African *Amanita muscaria*

Historical records of *Amanita muscaria* s.l. from South Africa (from before 1945) were obtained from (Doidge, 1950). To compare the historical records with the current distribution of the fungus, we downloaded all publicly available South African records from the Global Biodiversity Information Facility (GBIF) on 24 July 2023 (GBIF, 2023). We looked at the photographs linked to each record to identify surrounding tree species. Because every record suggested an association of *A. muscaria* with *Pinus* spp., we extracted information about plantation forestry land cover within South Africa from the 2022 South African National Land Cover dataset (Department of Forestry, Fisheries and Environment). We mapped the historical and current records of *A. muscaria*. We note that historical records often use older place names for both the country and its provinces, for example Transvaal for Gauteng or Mpumalanga.

### Mushroom collections

We sequenced genomes from a total of 24 *A. muscaria* and 1 *Amanita pantherina* mushrooms collected from South Africa, Australia, Europe and North America (Table 1). South African (*n =* 9) and Australian (*n =* 2) specimens were collected from pine plantations while European *A. muscaria* (*n =* 11), *A. pantherina* (*n =* 1) and Californian (*n =* 2) mushrooms were collected from native forests (Table 1; Supporting Information Fig. S1). Specimens were dried within 1–2 d using conventional food driers at low heat (less than 35°C). Our sampling scheme was designed to contextualize South African *A. muscaria* by integrating new data within published phylogenies, primarily Geml *et al*. (2008); a comprehensive survey of the global species complex was beyond our scope. We targeted as many South African specimens as possible, European specimens from three countries (based on what we found and could collect), and other specimens from additional clades identified by Geml *et al*. (2008), for example Clade I, represented by our two Californian mushrooms. Out of curiosity, we also sequenced two Australian specimens. Before sequencing, all specimens were stored as dried mushrooms at room temperature in either the Pringle or Vilgalys Laboratory fungaria at the University of Wisconsin–Madison and Duke University, respectively.

**Table 1:** Collection metadata for collected mushrooms.

Specimen ID	Collection date	Location	Region	Country	Latitude	Longitude	Species	Habitat	Host	Collector
11662	27 February 2019	Bulwer	KwaZulu‐Natal	South Africa	29.857681 S	29.710906 E	*A. muscaria*	Plantation	*Pinus patula*	J. Roux
11663	27 February 2019	Bulwer	KwaZulu‐Natal	South Africa	29.857681 S	29.710906 E	*A. muscaria*	Plantation	*Pinus patula*	J. Roux
11664	27 February 2019	Bulwer	KwaZulu‐Natal	South Africa	29.857681 S	29.710906 E	*A. muscaria*	Plantation	*Pinus patula*	J. Roux
11665	6 March 2019	Bulwer	KwaZulu‐Natal	South Africa	29.857681 S	29.710906 E	*A. muscaria*	Plantation	*Pinus patula*	J. Roux
11666	25 April 2019	Kranskop /Greytown	KwaZulu‐Natal	South Africa	N/A	N/A	*A. muscaria*	Plantation	*Pinus elliottii*	I. Greyling
11667	25 April 2019	Kranskop /Greytown	KwaZulu‐Natal	South Africa	N/A	N/A	*A. muscaria*	Plantation	*Pinus elliottii*	I. Greyling
11668	25 April 2019	Kranskop /Greytown	KwaZulu‐Natal	South Africa	N/A	N/A	*A. muscaria*	Plantation	*Pinus elliottii*	I. Greyling
11669	25 April 2019	Kranskop /Greytown	KwaZulu‐Natal	South Africa	N/A	N/A	*A. muscaria*	Plantation	*Pinus elliottii*	I. Greyling
11670	25 April 2019	Kranskop /Greytown	KwaZulu‐Natal	South Africa	N/A	N/A	*A. muscaria*	Plantation	*Pinus elliottii*	I. Greyling
Roed_3	12 September 2019	Østfold	Østlandet	Norway	59.4237 N	10.5937 E	*A. muscaria*	Native habitat	Not recorded	S.L. Harrow
Sogn_5	15 September 2019	Oslo	Østlandet	Norway	59.9822 N	10.7415 E	*A. muscaria*	Native habitat	Not recorded	S.L. Harrow
Skrap_3	17 September 2019	Oslo	Østlandet	Norway	59.8657 N	10.8562 E	*A. muscaria*	Native habitat	Not recorded	S.L. Harrow
Nes_1	17 September 2019	Akershus	Østlandet	Norway	59.8666 N	10.5181 E	*A. muscaria*	Native habitat	Not recorded	S.L. Harrow
Frag_1	18 September 2019	Oslo	Østlandet	Norway	59.9820 N	10.6742 E	*A. muscaria*	Native habitat	Not recorded	S.L. Harrow
Ring_1	6 October 2019	Zurich	Zurich	Switzerland	47.3617 N	8.4853 E	*A. muscaria*	Native habitat	Not recorded	S.L. Harrow
Wirz_3	7 October 2019	Nidwalden	Nidwalden	Switzerland	46.9053 N	8.3696 E	*A. muscaria*	Native habitat	Not recorded	S.L. Harrow
Gril_1	7 October 2019	Nidwalden	Nidwalden	Switzerland	46.9010 N	8.3571 E	*A. muscaria*	Native habitat	Not recorded	S.L. Harrow
Kara_3	7 October 2019	Zurich	Zurich	Switzerland	47.3617 N	8.4853 E	*A. muscaria*	Native habitat	Not recorded	K. O'Keefe
Nagy_Heves_A	12 October 2013	Heves	Heves	Hungary	47.317313 N	19.941566 E	*A. muscaria*	Native habitat	*Picea abies or Betula pendula*	L. Nagy
Nagy_Heves_B	12 October 2013	Heves	Heves	Hungary	47.317313 N	19.941566 E	*A. muscaria*	Native habitat	*Picea abies or Fagus sylvatica*	L. Nagy
20031	17 December 2021	Point Reyes National Seashore	California	United States	38.0519 N	122.8310 W	*A. muscaria*	Native habitat	Not recorded	Wang/Pringle
20045	19 December 2021	Point Reyes National Seashore	California	United States	37.9702 N	122.7304 W	*A. muscaria*	Native habitat	Not recorded	Wang/Pringle
NzAUS95	27 May 2019	Railton	Tasmania	Australia	41.375431 S	146.385042 E	*A. muscaria*	Plantation	*Pinus radiata*	Vilgalys/Henderson/Uehling
Aus332	31 Mar 2018	Penrose	NSW	Australia	34.630017 S	150.211625 E	*A. muscaria*	Plantation	*Pinus radiata*	Vilgalys/Henderson/Uehling
Nes_pan3	17 September 2019	Akershus	Østlandet	Norway	59.8666 N	10.5181 E	*A. pantherina*	Native habitat	Not recorded	S.L. Harrow

### Genome extraction, sequencing, assembly, annotation and quality control

#### DNA extraction and sequencing

High molecular weight DNA (HMW‐DNA) was extracted from samples of dried mushroom tissues using previously published protocols involving a solution of 25 : 24 : 1 phenol : chloroform : isoamyl alcohol (Bok *et al*., 2005; Nickles *et al*., 2023). Sequencing was performed with an Illumina NovaSeq 6000 platform with the aim of obtaining 6 million reads per sample to achieve a coverage of 30× or higher. We estimated the genome size as 44 Mb.

#### Genome assembly, annotation and quality analyses

Raw sequencing data were trimmed with Trimmomatic v.0.36 (Bolger *et al*., 2014) with LEADING:20 TRAILING:20 SLIDINGWINDOW:4:25 MINLEN:50, and genomes were assembled with SPAdes v.3.11.1 (Bankevich *et al*., 2012; Prjibelski *et al*., 2020) using default parameters. Assemblies were filtered to keep only the contigs/scaffolds larger than 500 bp. Assembled genomes were annotated with Augustus v.3.4.0 using models prebuilt for *Laccaria bicolor* (Stanke *et al*., 2008; Hoff & Stanke, 2013). Genome statistics of each genome were generated using seqtk (Li, 2012) and Benchmarking Universal Single‐Copy Orthologs (BUSCO; Simao *et al*., 2015) completeness was determined using Busco v.3.0.2 with the agaricales_odb10 database (Manni *et al*., 2021).

Before examining the assembled genomes, basic assembly metrics were determined using seqtk. To screen for potential fungal contamination, duplicate BUSCO genes were identified with BUSCO (v.3.0.2) run in genome mode using the Fungi Odb10 database with default settings. Only one genome showed a single duplicated BUSCO gene, suggesting all genomes were free from fungal contaminants. The first 200 nucleotides of each contig from each fully assembled genome were then used to query NCBI's prokaryote nucleotide database (accessed on 27 July 2023) in a local BLASTn search with default settings using the NCBI's Blast+ software package (v.2.6.0; Altschul *et al*., 1990; McGinnis & Madden, 2004). Contigs with matches showing an e‐value less than 1e‐5 were considered contaminated and removed from each genome. Genome quality metrics were then recomputed with seqtk. The decontaminated genomes were used for all subsequent analyses (Dataset S1).

#### Generating and filtering SNP predictions

Short reads were quality controlled with BBMap v.38.32 (Bushnell, 2015) and aligned to the NCBI reference genome (accession GCA_001691765.1) using Bwa mem v.0.7.17 (Li & Durbin, 2009). Variants were called using Genome Analysis Toolkit v.4.0.12.0 (McKenna *et al*., 2010) using parameters and hard filters identical to Drott *et al*. (2023). Single‐nucleotide polymorphism (SNP) data were filtered using VCFtools (v.0.1.13; Danecek *et al*., 2011), such that only biallelic sites were retained. Split decomposition was employed to reconstruct the phylogenetic tree using the filtered SNP dataset to identify potential errors or artifacts in the dataset (Methods S1).

### Clone correction and population genetics

#### Clone correction

To test whether different specimens are clones of each other, that is mushrooms generated from a single mycelium, or are distinct genetic individuals, we estimated pairwise kinships using KING‐robust (Manichaikul *et al*., 2010) using the function snpgdsIBDKING implemented in the R library SNPRelate.

#### Genetic differentiation and estimation of gene flow

Pairwise *F*‐statistics (*F*_ST_) and Euclidian distances (*D*) between individuals as well as the identification of genetic variants and private alleles between and within population were calculated or undertaken using the dartR package in R (Gruber *et al*., 2018). Tajima's *D* was estimated in 5000‐bp sliding windows using VCFtools (v.0.1.13; Danecek *et al*., 2011) with a subset of Clade II genomes from Europe and South Africa. Nucleotide diversity (π) was calculated across the entire genome using pixy (Korunes & Samuk, 2021) and heterozygosity was calculated across all variant sites using VCFtools.

### Phylogenomic analyses

#### Nomenclature of the *A. muscaria* species complex and closely related species and downloading of publicly available *A. muscaria* gene markers

We contextualized newly sequenced genomes using sequences of the internal transcribed spacer (ITS), ꞵ‐tubulin (β‐tub), nuclear large ribosomal subunit (LSU) and translation elongation factor 1‐alpha (TEF‐1⍺) genes from three key publications (Oda *et al*., 2004; Geml *et al*., 2008; Vargas *et al*., 2019; Dataset S2). Specimen ID codes from Oda *et al*. (2004) were shortened to only include the numeric ID. The taxonomy and nomenclature of *A. muscaria* s.l. are complex, and we used published literature to develop naming protocols.

Following recent precedent (Vargas *et al*., 2019), we used Geml *et al*.'s (2008) clade labels to identify our specimens. While taxonomists used to consider *A. muscaria* as a complex of subspecific varieties existing under a single species concept and identifiable on the basis of cap color (Miller & Jenkins, 1978), when specimens with different cap colors were integrated into single phylogenies using DNA sequence data, new species concepts emerged (Oda *et al*., 2004; Geml *et al*., 2006). Geml *et al*. (2008) represent the last major phylogenetic revision of the taxa, but instead of naming clades as species, Geml *et al*. (2008) chose to label distinct clades with roman numerals. We use the same numbering system for our specimens and for the specimens associated with already published data (Oda *et al*., 2004; Geml *et al*., 2008; Vargas *et al*., 2019), with two exceptions: after Geml *et al*. (2008) was published, *A. persicina* was formally recognized as a species, and we use its name (Tulloss *et al*., 2015). We also use the name *A. regalis* because it was formally published in a previous revision of the species complex (Neville & Poumarat, 2004) and is supported by the later phylogenetic evidence (Geml *et al*., 2008).

We used the same logic to identify specimens associated with other public data. Any specimen originally classified as and submitted to GenBank as one of the widely recognized and currently accepted infraspecific taxa of *A. muscaria* was given the working designation *A. muscaria* s.l. and later identified using Geml *et al*.'s (2008) clade labels. For our purposes, *A. muscaria* s.l. includes *A. muscaria* var. *guessowii*, *A. muscaria* var. *muscaria* and *A. muscaria* var. *flavivolvata*. However, if a specimen had a GenBank accession for ITS locus sequence data and was submitted as *Amanita regalis* (or *A. muscaria* var. *regalis*) or *Amanita persicina* (or *A. muscaria* var. *persicina*), we used the modern nomenclature (Tulloss & Yang, 2010), in other words the names *A. regalis* or *A. persicina*. These two taxa are easily differentiated from *A. muscaria* s.l. on the basis of gross morphology. Specimens classified as *A. pantherina* were kept as such. For the two specimens lacking an ITS accession, the metadata associated with the LSU accession was used instead. All collection location data were extracted from the publication corresponding to each given collection.

#### Species tree construction using multi‐loci barcodes

We aligned ITS (*n* = 189), β‐tub (*n* = 102), LSU (*n* = 98) and TEF‐1⍺ (*n* = 52) sequences with Mafft v.7.511 (Katoh & Standley, 2013) with an automated model selection (L‐INS‐i, FFT‐NS‐i and FFT‐NS‐2) and adjustment of reverse complements. Alignments were trimmed with Trimal v.1.4.rev15 (Capella‐Gutierrez *et al*., 2009) with the gappyout parameter. Once trimmed, alignments for all four regions were used to construct a maximum likelihood phylogeny using an edge‐linked partition model with Iq‐Tree multicore v.2.2.0 (Minh *et al*., 2020), with the optimal substitution model determined by Modelfinder (Kalyaanamoorthy *et al*., 2017) and 1000 rapid bootstraps.

### Specialized metabolite biosynthetic gene cluster prediction and characterization

#### Genome mining for specialized metabolite BGCs

To identify canonical SM biosynthetic gene clusters (BGCs; i.e. those with experimentally vetted class‐defining core biosynthetic genes (termed backbone genes), e.g. polyketide (PK) BGCs, which are a canonical class of SM defined by polyketide synthase (PKS) backbone genes). Fungal antiSmash (v.5; Blin *et al*., 2019) was run on all of the genomes using the default settings. However, specimen 11 662 was erroneously omitted from genome mining analyses. Closely related terpene or PKS BGCs were networked into gene cluster families (GCFs) using BiG‐Scape v.1.0.1 (Navarro‐Munoz *et al*., 2020). An optimal networking cutoff of 0.3 was determined across a testing range of 0.1 to 0.6 (Figs S2, S3).

We ran cblaster (v.1.3.18; Gilchrist *et al*., 2021) using a previously characterized ibotenic acid BGC sequence as the query to identify the noncanonical ibotenic acid BGC (Obermaier & Müller, 2020), which is made up of the genes (from left to right) *iboA*, *iboF*, *iboD*, *iboC*, *iboG1*, *iboH* and *iboG2*. We further investigated the distribution of genes in the ibotenic acid BGC using reciprocal best‐hit blast analysis. Briefly, characterized protein sequences were used to replace corresponding protein predictions of IboA, IboC, IboD, IboF, IboG1, IboG2 and IboH (Kohler *et al*., 2015) in the file containing the proteome of mushroom Gril_1. The resulting proteome was queried against a dataset of all annotated publicly available protein sequences downloaded from NCBI in the spring of 2023 with methods similar to those described previously (Drott *et al*., 2020). Any two hits that were within 30 kb of each other were considered physically clustered. The presence of a putative ortholog was mapped to a modified version of the whole‐kingdom phylogeny from Nickles *et al*. (2023) using ggtree (Yu *et al*., 2017).

We searched for the presence of amatoxin‐encoding ‘MSDIN’ genes and the toxin‐processing *popB* gene using methods and publicly available scripts from (Drott *et al*., 2023), as detailed in Methods S1.

#### Phylogeny of Agaricomycete polyketide synthase proteins

A representative query protein was selected from the two conserved PKSs found within the *A. muscaria* genomes. Each genome's protein was Blastp (Altschul *et al*., 1990; Schaffer *et al*., 2001) searched against every annotated Agaricomycetes genome publicly available on NCBI as of 1 December 2022 (*n =* 292 genomes) with an e‐value cutoff of 1e^−5^. Only the top hit for each BLAST search was retained for further analysis. A gene tree of resulting sequences was constructed with the same methodology used to construct the species tree. To confirm whether the observed PKS duplication was unique to the *A. muscaria* species complex, we also conducted a targeted analysis of the *Amanita* genus. Genes encoding all PKS enzymes were identified from the six available non‐*muscaria Amanita* genomes. The resulting 18 PKS protein sequences were used to construct a gene tree with the same methodology described to construct the species tree. Domain analysis revealed a single hybrid nonribosomal peptide synthetase (NRPS)‐PKS protein in *A. inopinata* (KAF8636077.1), which was subsequently used to root the tree.

### Metabolomics for chemical identification of metabolites

#### Methanolic extraction of metabolites

We extracted metabolites from nearly every dried mushroom that had its genome sequenced (*n* = 22); the two Australian specimens (Aus332 and NzAUS95) were not included in metabolomics because there were insufficient tissues left. Two additional North American (20031‐a and 20 045‐b) and one additional South African (11671) mushrooms whose genomes were not sequenced were assayed, bringing the total number of metabolite extracts to 26 (*A. muscaria*: 25 and *A. pantherina*: 1; Table S1).

All samples were imaged before extraction (Fig. S1). Mushroom caps were pulverized and extracted overnight in 20–100 ml (depending on the volume of the mushroom tissue) of high‐performance liquid chromatography (HPLC)‐grade methanol. Extracts were filtered with a 0.2 μm syringe filter and evaporated to dryness on a rotary evaporator. Compounds were resuspended at 1 mg ml^−1^ in methanol or dimethylsulfoxide (DMSO) for chemical analysis or biological assays, respectively.

#### UHPLC–MS/MS analysis

Mushroom cap extracts were characterized on Ultra‐high‐performance liquid chromatography coupled with tandem mass spectrometry (UHPLC–MS/MS) using only spectroscopic grade solvents. Data were acquired using a Thermo Fisher Scientific Q Exactive Orbitrap mass spectrometer (Waltham, MA, USA) coupled to a Vanquish UHPLC (Waltham, MA, USA) operated in positive ionization mode. For all runs, we used a Waters XBridge BEH‐C18 column (2.1 mm × 100 mm, 1.7 μm) and added 0.05% formic acid to our spectroscopic grade acetonitrile and water (flow rate 0.2 ml min^−1^). The 37 min screening gradient method for the samples is as follows: Starting at 10% organic for 5 min, followed by a linear increase to 90% organic over 20 min, another linear increase to 98% organic for 2 min, holding at 98% organic for 5 min, decreasing back to 10% organic for 3 min, and holding at 10% organic for the final 2 min.

#### Global Natural Product Social Molecular Networking (GNPS) analysis

To construct a molecular network, the raw mass spectra were converted to the mzXML format using the RawConverter software (v.1.2.01., The Scripps Research Institute; He *et al*., 2015). We used the web‐based server to run a GNPS analysis with the default parameters provided by the platform: Min pairs cos = 0.7, network TopK = 10, max connected components size = 100, min matched fragment ions = 6, min cluster size = 2 (Wang *et al*., 2016). A molecular network was created with a precursor ion mass tolerance of 2.0 Da and fragment ion mass tolerance of 0.5 Da. The resulting molecular network was visualized in Cytoscape (v.3.10.2.; Shannon *et al*., 2003).

#### Preparing metabolites for bioactivity screening

Every crude extract was tested against a range of laboratory model organisms available to us at the University of Wisconsin–Madison. Organisms included the pathogenic bacterium Methicillin‐resistant *Staphylococcus aureus* (MRSA), *Pseudomonas aeruginosa* and the pathogenic fungus *Candida auris*. Because experiments involving invertebrates are more complex, crude extracts from only a subset of the samples were used in tests against the nematodes *Caenorhabditis elegans* and *Brugia* spp., and the dipterans *Aedes aegypti* and *Musca domestica*. For nematode screening, we used three extracts from each region (United States, South Africa and Europe) in addition to extracts from the outgroup, *A. pantherina*. For insect screening, we used one sample from each of the three regions in addition to the outgroup (Table S1). Crude extracts were redissolved in methanol (MeOH) to test for antimicrobial activity or DMSO for invertebrate assays. Details of bioactivity assays are provided in Methods S1. Our focus on model organisms was a practical choice and enables basic insights into bioactivity; future tests with species and populations of South African antagonists are needed.

## Results

### South African *A. muscaria* derive from a single European species

*Amanita muscaria* has long been associated with imported pines (Box 1; Fig. 1), and its history in South Africa is remarkably well recorded. The earliest herbarium specimen dates to 1874 (Box 1), but historical records suggest the fungus was already established by then. Today, it is common in planted forests, cities and on rural farms (Fig. 1).

**Fig. 2:**
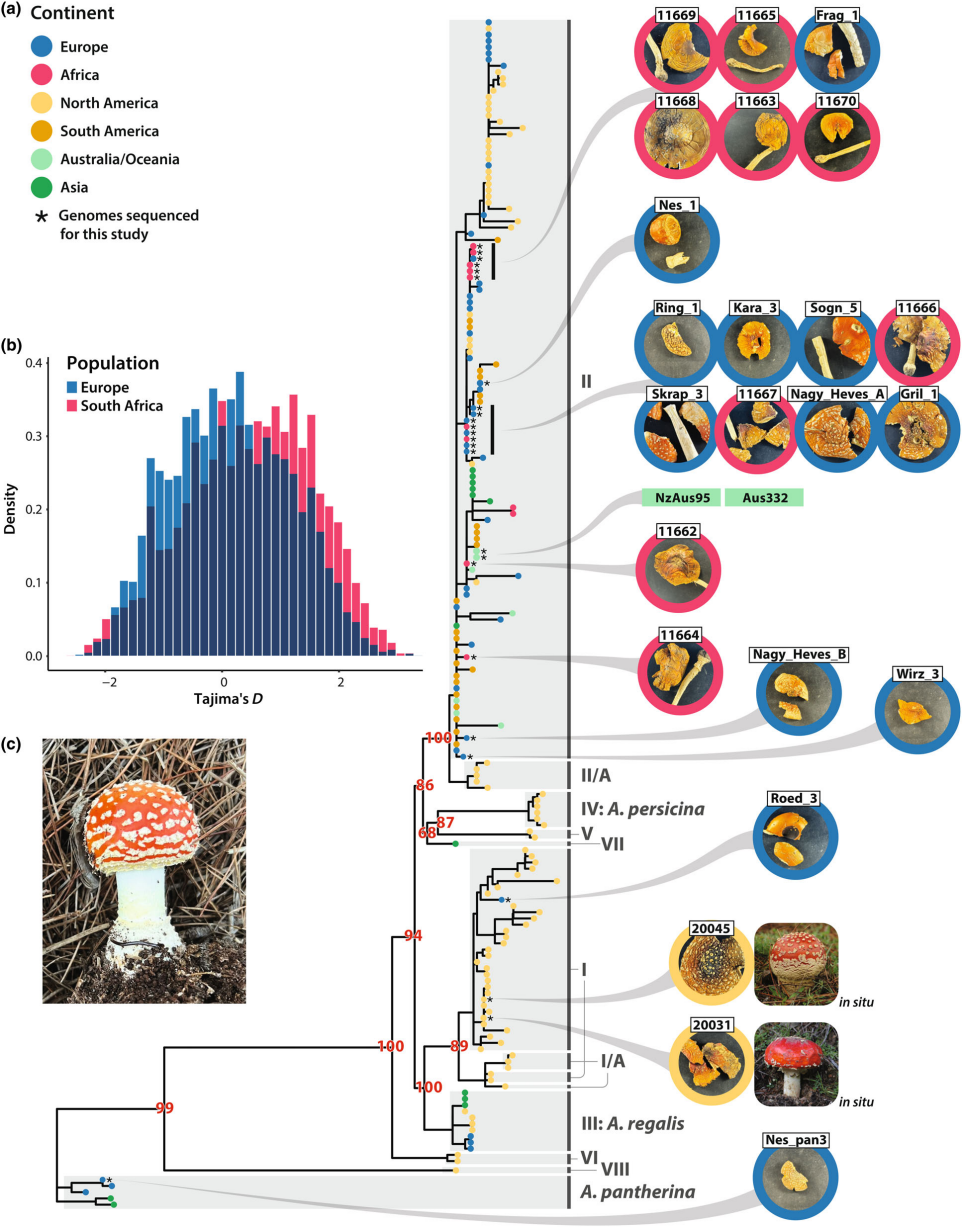
Phylogenetic species reconstruction of 184 *Amanita muscaria* and 5 *Amanita pantherina* mushrooms. (a) Phylogeny constructed from an edge‐linked partition model of ITS, LSU, β‐tub and TEF‐1⍺ gene sequences. Clades are labeled with either the numbers used in Geml *et al*. (2008) or as *Amanita persicina* or *Amanita regalis*; many of the labels from Geml *et al*. (2008); e.g. clade VIII) represent undescribed taxa. Our phylogeny groups two specimens from Clade I in Geml *et al*. (2008) with Clade I/A. The tree is rooted with *A. pantherina*. Bootstrap support values are shown in red. Each mushroom's continent of origin is indicated by tip color, and available images of our sequenced mushrooms are displayed to the right of the phylogeny. Colors of the circles surrounding individual images mark the mushroom's continent of origin. (b) The distribution of Tajima's *D* was estimated from 5000‐bp windows across the reference genome using biallelic single‐nucleotide polymorphisms (SNPs) from the European (blue) and South African (red) Clade II genomes sequenced by us. (c) A photograph of *A. muscaria* collected near pine trees on a golf course (Simola Golf and Country Estate) in 2023 in the Western Cape province of South Africa.

To investigate the origin of South African *A. muscaria*, we reconstructed phylogenies of *A. muscaria* using data from our own genomes and public repositories (Dataset S2); the topologies of created trees were largely in agreement with minor differences (Methods S1; Figs S4, S5). The nine South African individuals we sequenced belong to Clade II of the *A. muscaria* complex (Fig. 2a), a lineage also including specimens from Australia and South America, where *A. muscaria* is also introduced and spreading (Vargas *et al*., 2019). Clade II broadly corresponds to *A. muscaria var. muscaria*, widespread across Eurasia and into Alaska.

Site‐frequency spectrum analyses support a historical introduction of *A. muscaria* into South Africa. European Clade II individuals show a near‐zero value (Fig. 2b), while South African individuals display a positive shift, consistent with the loss of rare alleles during a founder event. The European mushrooms consistently have higher nucleotide diversity (0.00166) and mean heterozygosity (0.0799 ± 0.0132; Table S2) as compared to the South African population (0.00118 and 0.0684 ± 0.0045, respectively). Although sampling differences might influence results, the results align with historical records and phylogenetic inferences. Additionally, although the South African population shows signs of a bottleneck, the population remains diverse and is sexual: no clones were detected in either population and kinships are typically below 0.2 (Dataset S3). However, one pair of mushrooms from a single *P. elliottii* plantation in KwaZulu‐Natal has a higher kinship (*c*. 0.33), suggesting some level of inbreeding, perhaps between multiple monokaryotic mycelia of a shared parent.

Roed_3 was the only European individual we sequenced falling into Clades I‐I/A. Clades I‐I/A represent a complex of two cryptic taxa widely distributed throughout North America. Interestingly, mitochondrial DNA sequences placed Roed_3 closer to Clade II than Clades I‐I/A, a pattern we speculate as reflecting a hybridization event (Figs S4, S5).

### Specialized metabolite gene clusters are conserved across geographic regions and phylogenetic clades

**Fig. 3:**
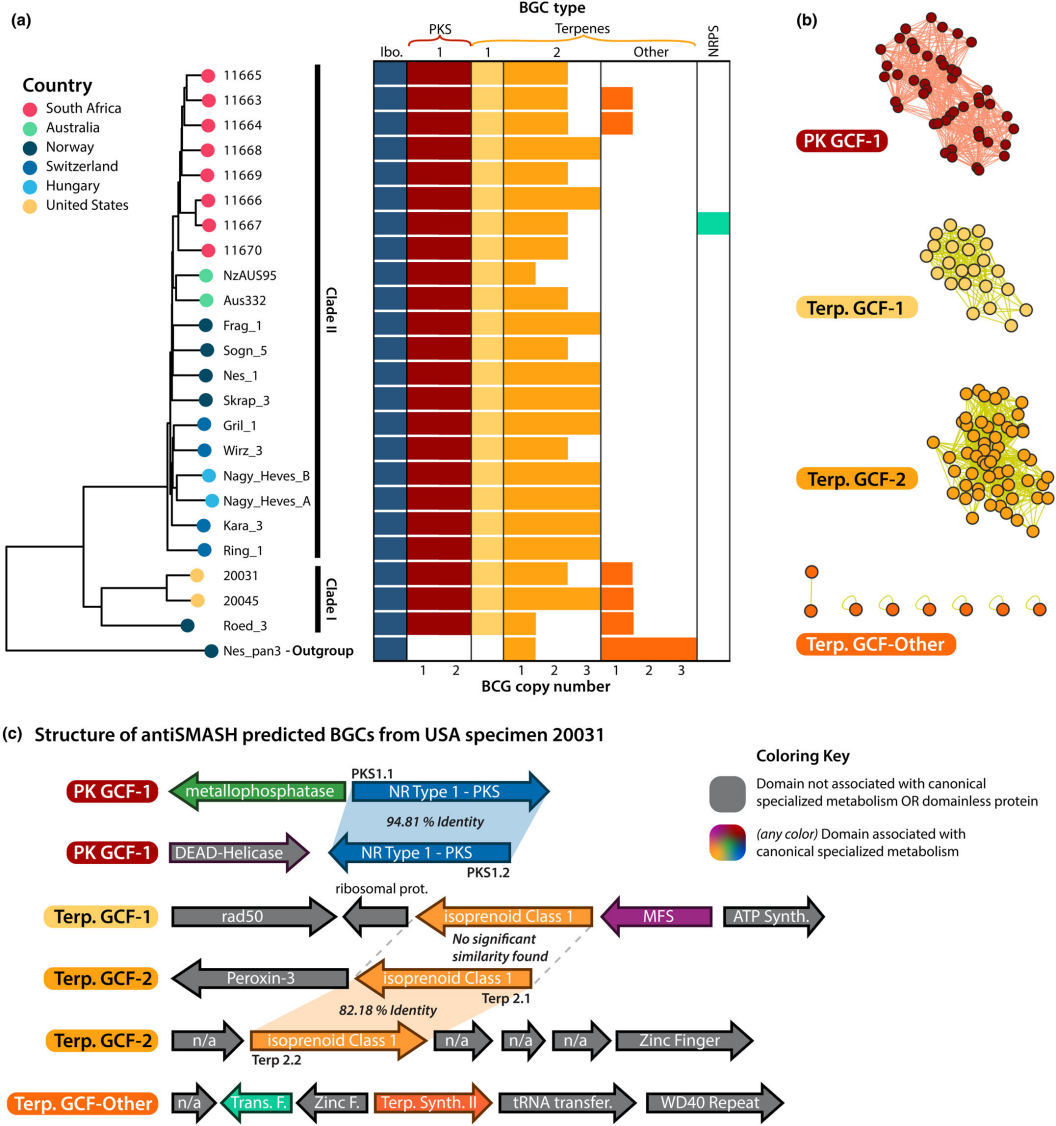
Specialized metabolism (SM) in *Amanita muscaria* genomes. (a) Numbers of biosynthetic gene clusters (BGCs) per genome, sorted by type based on backbone enzymes. Gene copy numbers for each BGC locus also depicted. The maximum likelihood phylogeny of the isolates was generated using single‐nucleotide polymorphism (SNP) data and rooted with *A. pantherina* (Nes_pan3). The profiles of our South African genomes appear similar to the profiles of our European genomes. (b) Gene cluster family (GCF) networks of the two major SM classes within the genomes: polyketide synthase (PKS) and terpenes (Terp.). Nodes represent a single BGC from an individual genome and edges between nodes connect related nodes. Networked nodes represent closely related clusters likely producing the same or similar products (i.e. a GCF). (c) Domain and genetic architecture of the antiSMASH‐predicted BGC loci using Californian sample 20 031‐a (Clade I) as an example; the panel illustrates the unique architectures of each GCF. Genes are depicted approximately to scale. All major GCFs were found in similar copy numbers across *A. muscaria* populations, suggesting the retention of chemical potential among groups.

AntiSMASH predicted between six and eight BGCs per genome, all of which were Type‐1 PKSs or isoprenoid(s) biosynthesis (terpene) backbone enzymes, with the exception of a single NRPS found in the South African specimen 11 667. All genomes, including the *A. pantherina* outgroup, contained the full ibotenic acid BGC (Figs 3a, S6, S7) as defined previously (Obermaier & Müller, 2020). While the products of most of these *A. muscaria* BGCs are unknown, we confirmed the ibotenic acid BGC produced muscimol in each of the *A. muscaria* mushrooms we sequenced, regardless of phylogenetic grouping or geographic origin. Muscimol was also made by the *A. pantherina* outgroup (Nes_pan3 in Fig. S8).

BGCs were networked into the GCFs (Figs 3b, S2, S3) likely to produce identical or closely related SMs (Navarro‐Munoz *et al*., 2020; Bağcı *et al*., 2025). All *A. muscaria* genomes encoded two PKSs, with both copies (referred to as PKS1.1 and PKS1.2; Fig. 3c) grouping into the same GCF, termed PK GCF‐1. The terpene BGCs, which showed more variability in copy number, were grouped into two major GCFs termed terpene GCF‐1 and terpene GCF‐2. Terpene GCF‐1 was universally present as a single copy in every *A. muscaria* genome but was absent in the outgroup. By contrast, the copy number of terpene GCF‐2 varied from 1 to 3 across samples, with no synteny observed among the neighboring BGC genes (Fig. 3c). The final group, termed terpene GCF‐‘other’ (Fig. 3), represents all terpenes whose predicted BGC locus was found only in a single isolate. Variation in terpene gene copy number may reflect genome fragmentation or incomplete assemblies. However, expansions of terpenes are common across the Basidiomycota. A thorough analysis of these BGCs is beyond our scope but emerges as an interesting direction for future research. Only a small fraction of genes in antiSMASH‐predicted BGCs housed domains associated with SM in model Ascomycetes, suggesting a different biosynthetic logic in *A. muscaria* – either through stand‐alone backbone enzymes or from the recruitment of novel tailoring genes.

Although *A. muscaria* has been reported to produce lethal amatoxins (Faulstich & Cochet‐Meilhac, 1976), we found no genomic or chemical evidence – using bioinformatic searches, relaxed BLAST queries and direct metabolite analysis – to support these claims (see Fig. S1).

### Conserved PKS enzymes in *A. muscaria* originated from duplication in a common ancestor of the species complex

**Fig. 4:**
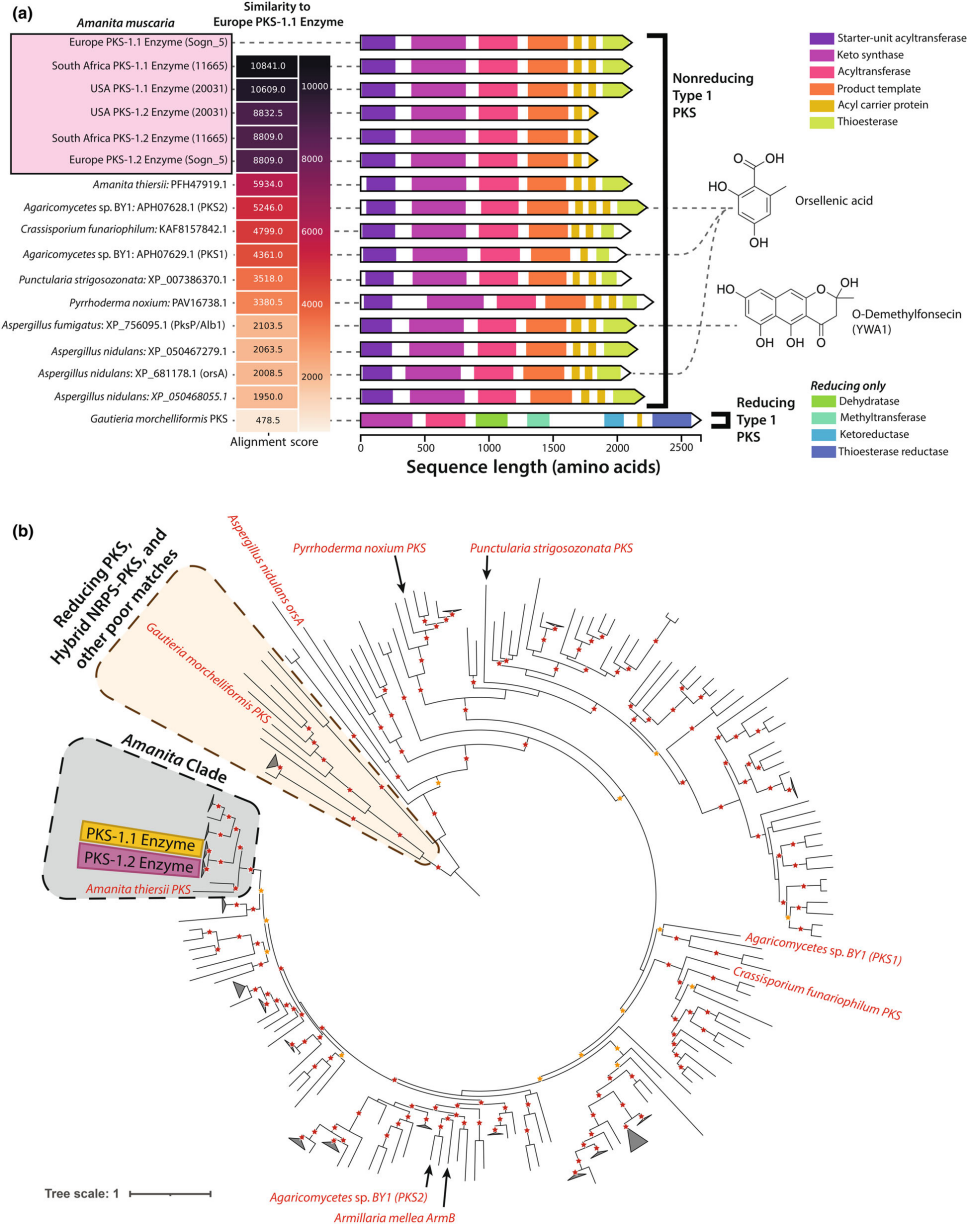
Analysis of the Type 1 polyketide synthase (PKS) gene duplication found in *Amanita muscaria*. (a) Comparison of the two Type I PKS enzymes found in the PK GCF‐1 gene cluster family (termed PKS‐1.1 and PKS‐1.2), including copies from European, South African and Californian *A. muscaria* genomes (highlighted in pink box). Left to right: Similarity scores were calculated by aligning each PKS sequence to the Sogn_5 PKS‐1.1 backbone enzyme. The domain architecture of each PKS is color‐coded. Known metabolites associated with specific PKSs are linked by dashed lines to the corresponding structures on the right. (b) Midpoint‐rooted phylogeny of 244 Agaricomycete PKS proteins with additional hand‐selected PKS proteins (e.g. reducing and nonreducing PKSs, *orsA* from *Aspergillus nidulans*). The tree was midpoint‐rooted to facilitate clade visualization. Clades with an average branch length of less than 0.3 to their leaves were collapsed and displayed as triangles sized proportionally to the number of collapsed leaves. The monophyletic clade containing all *Amanita* PKS proteins is shaded in gray. A detailed analysis of proteins from the genus is provided in Supporting Information Fig. S9. The clades for *A. muscaria* PKS‐1.1 and PKS‐1.2 are highlighted in yellow and purple, respectively. Nodes with > 80% and > 90% support are indicated with orange and red stars, respectively. A subset of the proteins used to build the phylogeny are labeled for reference.

The strong similarity between the two copies of PKS enzymes in all *A. muscaria* genomes led us to hypothesize the copies arose from a recent duplication event. In a phylogenetic analysis, all of *A. muscaria*'s PKS sequences form a monophyletic clade, while all other PKS sequences from other *Amanita* spp. share more ancient ancestry (Fig. 4b). A targeted follow‐up analysis confirms the PKS duplication as unique to *A. muscaria* (Fig. S9). This analysis also revealed an interesting and distinct evolutionary history of the PKS enzyme in *A. inopinata*, a lineage which has independently duplicated the ancestral nonreducing PKS and acquired both a reducing PKS and a hybrid NRPS‐PKS (Fig. S9). The lack of *trans*‐species representation in the duplicated *A. muscaria* PKS clade is strong evidence for the duplication having occurred relatively recently, likely in a common ancestor of the *A. muscaria* species complex.

To test whether the duplication of PKS‐1.1 and PKS1.2 enabled functional divergence, we compared domain variation between the two copies and a sampling of highly similar PKS enzymes across diverse fungi. The PKS1.2 backbone in all *A. muscaria* genomes, regardless of geographic region or clade, has lost its C‐terminal thioesterase (TE) domain (Fig. 4a). This result is not an annotation error; we used tBLASTn to verify that the coding sequence for this domain was only present in the genomic DNA associated with PKS1.1. The C‐terminal domain is typically involved in the chain release and cyclization of PK products (Tang *et al*., 2019). While most PKS hits from closely related *Amanita* species were found in single copies within their genomes and retained the C‐terminal thioesterase, a distinct PKS clade – from *Amanita brunnescens* – showed a similar pattern of duplication and loss, suggesting the pattern has evolved multiple times in the genus (Fig. S9).

The domain structure of *A. muscaria*'s PKS enzymes closely mirrors the structure of known orsellinic‐acid‐producing PKSs in other Agaricomycetes (Lackner *et al*., 2013; Braesel *et al*., 2017) and in the Ascomycete *Aspergillus nidulans* (Schroeckh *et al*., 2009; Fig. 4a). While these findings may suggest *A. muscaria* is producing an orsellinic‐acid‐like compound, a similarly strong hit to a PKS in the *Aspergillus fumigatus* genome associated with production of a melanin intermediate (Fujii *et al*., 2000; Brakhage & Liebmann, 2005) raises some doubt about the true end‐product of the *A. muscaria* PKS enzymes.

### GNPS‐based untargeted mass spectrometry analysis suggests the *A. muscaria* metabolome is highly conserved across native and novel ranges

**Fig. 5:**
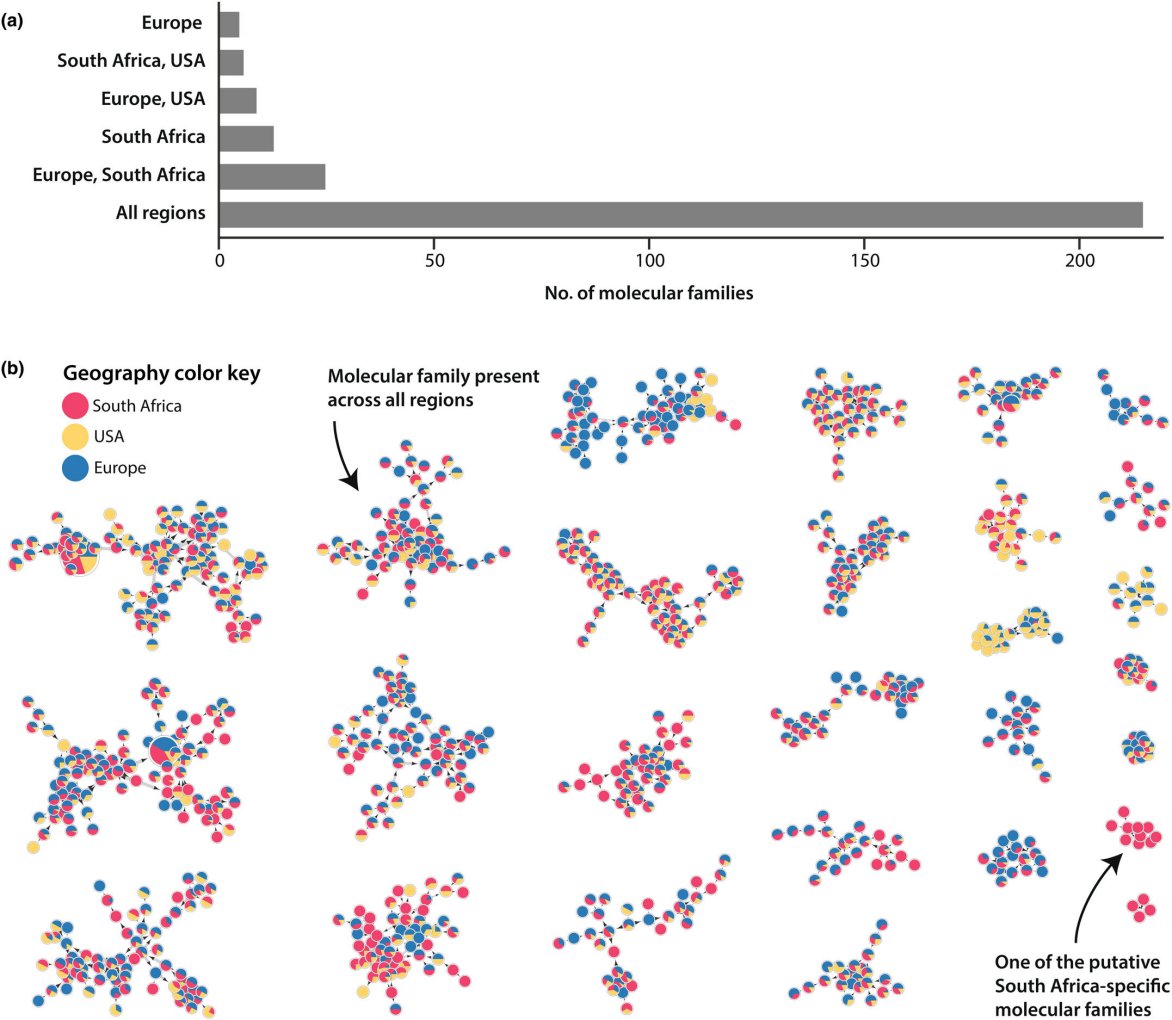
Metabolome of *Amanita muscaria* contextualized by geography. (a) Count of molecular families (MF) grouped by geographic region. (b) Network visualization of a subset of MFs. Node size reflects the total precursor intensity. Each node includes a pie chart marking the geographic regions where mushrooms with that MF were located. The full network of all detected MFs is in Supporting Information Fig. S10. Most MFs are found in all regions and comparatively few MFs are found only in South Africa.

GNPS analysis identified 273 unique molecular families (MFs) across all spectra (Fig. S10) including both primary and specialized metabolic features (Fig. 5a; see the Materials and Methods section). Of the 273 MFs, 215, including all MFs with at least 11 nodes, were detected in every geographic region. The largest MF (MF‐21), comprising 100 unique nodes, was predicted to belong to a primary metabolism phospholipid family. We identified 25 MFs unique to *A. muscaria* Clade II samples (South Africa and Europe) and 13 MFs specific to South African *A. muscaria*. The largest South Africa‐specific MF (MF‐9) was made up of 10 unique nodes (Fig. 5b). These nodes had precursor *m/z* values between 900 and 1200, with no matches to known library entries or previously characterized metabolites.

### *A. muscaria* extracts reveal biological activity/inactivity, with no population‐specific patterns

**Fig. 6:**
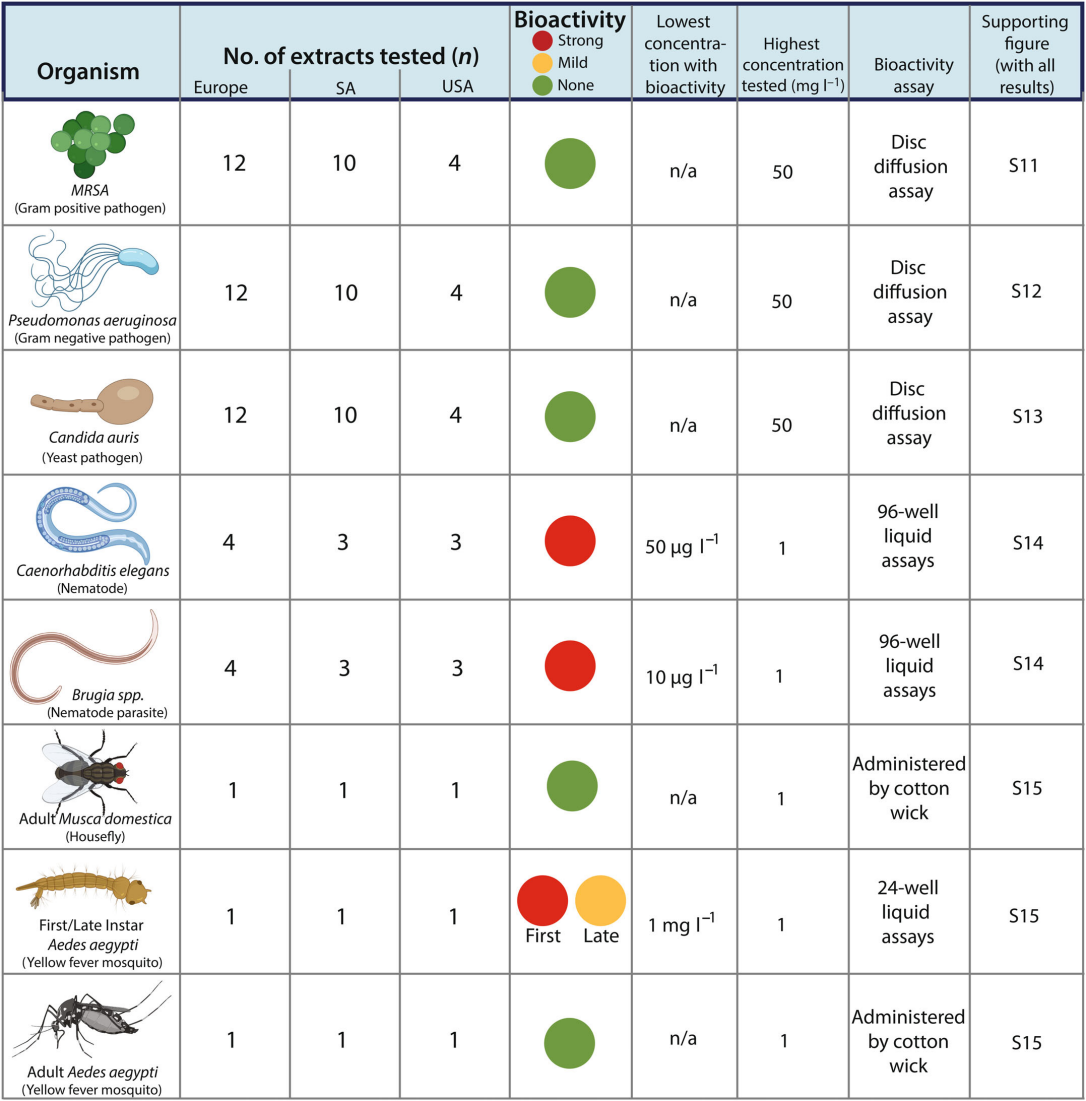
Bioactivities of *Amanita muscaria* extracts against Methicillin‐resistant *Staphylococcus aureus* (MRSA), *Pseudomonas aeruginosa*, *Candida auris*, *Caenorhabditis elegans*, *Brugia* spp., *Musca domestica* and *Aedes aegypti*. Columns from left to right are the organisms tested, number of extracts (mushrooms) tested in each experiment (SA = South Africa, USA = California), the viability of tested organisms when confronted with extracts, the concentrations used, the method of inoculation and a reference to Supporting Information with full results.

To understand the potential of *A. muscaria* metabolites to have broad‐spectrum activity against bacteria, filamentous fungi and yeasts, with potential relevance for ecological inferences and drug discovery, we performed disk‐diffusion assays against MRSA (Gram‐positive; Fig. S11), *Pseudomonas aeruginosa* (Gram‐negative; Fig. S12) and *Candida auris* (a *Saccharomyces* yeast; Fig. S13). None of these organisms were inhibited by extracts (Fig. 6). In contrast to the microbial assays, extracts from nine geographically representative *A. muscaria* samples and one *A. pantherina* sample all strongly inhibited the growth of the clade V nematode *Caenorhabditis elegans* (Figs 2, S14). Extracts also immobilized the Clade III parasitic nematodes *Brugia pahangi* and *Brugia malayi*, and assays with *B. malayi* reveal that the impact of extracts on *B. malayi* was similar to heat‐killed controls (Fig. S14). Insect assays revealed a more selective pattern: while adult *Musca domestica* and *Aedes aegypti* were unaffected by the extracts, first‐instar *A. aegypti* larvae were highly susceptible. In general, bioactivity declined as animals reached later developmental stages (Fig. S15). While the model organisms available to us may be unlikely to interact with *A. muscaria* in nature, our findings suggest there is variation in dipteran susceptibility, even despite the presence of GABA_A_ receptors in flies. Our data also suggest potential bioactivity may be limited to early developmental stages in certain species. We note we commonly find dipteran eggs laid among the gills of *A. muscaria* (Drott, Pringle and Stokes, pers. obs.). None of the bioactivities observed were different among the mushrooms collected from different continents. While our data suggest broad nematicidal activity, important differences may exist between the models we used and the populations of species interacting with *A. muscaria* in South Africa and elsewhere.

## Discussion

The mushrooms of the fungus *A. muscaria* s.l. are famous, featured in settings ranging from Super Mario™ to treatises of Hindu texts. Despite its prominent cultural status, the human‐mediated dispersal of this fungus is poorly documented. In this study, we used genomics and metabolomics to investigate the history and evolution of *A. muscaria* following its introduction to South Africa. Our phylogenetic analyses reveal South African *A. muscaria* populations belong to Clade II, a cryptic lineage of European origin. Historical records suggest the fungus was likely introduced more than 150 yr ago, perhaps with pine seedlings brought by European settlers to Cape Town. Its subsequent spread was facilitated by the expansion of pine forestry in southern Africa, and South African *A. muscaria* now grow with pines imported from other continents, including the Mexican *P. patula*. The fungus is sexual, and its populations in South Africa are genetically diverse, with multiple distinct individuals present within single forest stands. Despite its extended history in South Africa, the specialized metabolome of *A. muscaria* remains highly conserved, with gene clusters encoding muscimol, terpene and PK products present in all genomes. Bioactivity assays with laboratory model organisms reveal the potent nematicidal effects of the fungus's metabolites, suggesting their potential (as yet untested) role in mediating ecological interactions.

The European Clade II lineage found in South Africa is also the lineage introduced to Colombia (Vargas *et al*., 2019) and Australia (Lebel *et al*., 2024; Fig. 2a). Remarkably, the global dispersal of *A. muscaria* appears to involve just one of its many cryptic species (and we note many of the cryptic species await formal naming, e.g. the currently unnamed species of Clades I‐I/A (Geml *et al*., 2008), which include our two Californian genomes). Europe has long been suspected as the source of Australian *A. muscaria*, but we are the first to identify Clade II as the specific lineage introduced there. More intensive sampling will be needed to pinpoint the exact European source(s) of South African *A. muscaria*, and additional sequencing may reveal whether other clades (or after they are named, species) within the complex have been introduced elsewhere in the country. However, based on available data, Clade II appears to be the primary lineage driving invasions, suggesting underlying genetic, ecological or historical factors contributing to its spread.

The fungus may also have been introduced to South Africa from another invasive range, for example, Colombia or Australia. But historical records suggest the most parsimonious explanation for the *A. muscaria* in South Africa is a direct introduction(s) from Europe (Box 1; Fig. 1). Interestingly, several of our phylogenies (but not all) suggest South African *A. muscaria* share their most recent common ancestry with Australian populations, a pattern which may reflect a shared European source population (Figs S4, S5).

Kinship analyses indicate no two South African mushrooms are clones, although many mushroom pairs are closely related; the data are consistent with ongoing sexual reproduction. While invasive fungal populations often exhibit shifts in mating systems or reproductive modes (Wang *et al*., 2023), the conservation of sexual reproduction and mating type diversity in South Africa suggests either a weak genetic bottleneck or continued admixture from multiple introductions.

The introduction of *A. muscaria* into South Africa is also reflected in site‐frequency spectra, where a positive shift in Tajima's *D* estimates among Clade II mushrooms (Fig. 2b) is consistent with a founder event followed by insufficient time and/or evolution to regenerate rare alleles. A similar pattern has been observed in *A. phalloides* populations introduced to California from Europe (Drott *et al*., 2023). Other population genetic statistics, including Fst estimates, also suggest South African populations are differentiated from their European counterparts (Fig. S16a). Loci with the highest Fst scores encode genes associated with oxidoreductase activity, metal binding and cell membranes – functions previously linked to host and environmental stress responses in fungi (Fig. S16b; Staerck *et al*., 2018; Feng *et al*., 2023). Further research is needed to clarify the significance of the differences.

Despite the different ecological contexts of Europe, South Africa and Australia, all sequenced *A. muscaria* genomes housed a similar complement of BGCs, including the ibotenic acid BGC (Figs 3, S6, S7), an expanded set of terpenes and two PKS gene clusters (Fig. 4). Research on the ecology of *A. muscaria* has focused primarily on its ecological niche; the ECM symbiosis has one of its origins in the *Amanita* genus (Wolfe *et al*., 2012). Although little is known about the role of SMs in the ECM symbiosis, we identified an expansion of an orsellinic‐acid‐like PKS ortholog maintained across populations (Figs 3, 4; how the duplicate genes are expressed remains a target for future research). One of these orthologs has lost a TE domain, suggesting that the mature product is not cyclized or may depend on other enzymes for processing, similar to lovastatin biosynthesis (Xu *et al*., 2013). Orsellinic acid, found in several basidiomycetes (Lackner *et al*., 2012), has derivatives with potential herbicidal properties (Peres *et al*., 2009), which we speculate may influence fungal–plant interactions. Orsellinic‐acid‐sesterpene hybrids occur in some fungi, resulting in highly diverse bioactivities (Gao *et al*., 2023). *Trans*‐BGC interactions are increasingly being discovered (Won *et al*., 2022) but are not well understood, particularly in Basidiomycete lineages; we speculate that such epistatic interactions modulate the chemical diversity associated with the conserved terpene BGCs in *A. muscaria*.

The conservation of primary and SMs across introduced and native populations provides no evidence for relaxed selection (Fig. S17) as might be expected if metabolic resources were being reappropriated to other pathways (Blossey & Nötzold, 1995). Instead, our findings raise the possibility of conserved functional interactions with communities in both native and introduced ranges. Perhaps imported European soils harbored antagonistic microbes and invertebrates now interacting with *A. muscaria* in South Africa, or perhaps South African *A. muscaria* have equivalent kinds of interactions with native African antagonists. It is also possible there has not been enough time for genomic signatures of relaxed selection to emerge, although SMs are often subject to strong selection and are readily lost, causing their highly patchy distribution across taxa (Robey *et al*., 2021).

While *A. muscaria*'s core BGCs are highly conserved, we also identified 13 MFs unique to South African populations. Slight differences in mushroom handling after collection and differences among the microbial communities inhabiting mushrooms may have impacted identified metabolites, but we speculate these novel MFs represent metabolic innovations influencing interactions in the novel range. None of the consensus spectra of the South African MFs matched to public reference libraries in GNPS, additional evidence they represent truly novel metabolites evolving in the invasive range. The differentiation of metabolic space between fungal populations is often slight, but it can sometimes be defined by the production of a small subset of ecologically important metabolites (Drott *et al*., 2021).

The association between *A. muscaria* and fly‐killing dates back centuries, and both historical and modern accounts suggest insecticidal properties. The traditional recipe for using *A. muscaria* to kill flies involves soaking *A. muscaria* in milk or water (Lumpert & Kreft, 2016). As recently as 2021, Carboué & Lopez (2021) cited *A. muscaria* extracts as having fly‐killing bioactivity. However, skepticism about its ability to kill insects has persisted for just as long; as early as 1779, French botanist Jean Bulliard challenged claims of insecticidal activity and noted flies appeared unaffected by the mushroom (Wasson, 1969). Some folklore suggests *A. muscaria* may act as an attractant rather than a direct toxin, luring flies to consume the mushroom before they ultimately drown in the *A. muscaria*‐soaked milk.

Our findings align with historical skepticism – while *A. muscaria* extracts strongly suppressed nematodes (*C. elegans* and *Brugia* spp.) and inhibited mosquito larvae, they had no detectable effect on any adult dipterans (or bacteria or yeast; Fig. 6). Our experiments targeted model organisms, but the models serve as a robust proxy for detecting broad‐spectrum bioactivity. The strong nematicidal properties of *A. muscaria* reproduced by us have previously been attributed to its SMs ibotenic acid and muscimol (Johnston, 2014). Both are known agonists to the GABA_A_ receptor complex (Johnston, 2014). While we did not discover any effect on adult dipterans, muscimol resistance has been documented among mycophagous species of dipterans but not their frugivorous counterparts (Tuno *et al*., 2007), emphasizing questions about the susceptibility of relevant antagonists in the introduced range. Our experiments used model organisms as tools, but with these data, future efforts focused on South African antagonists can take a more targeted and informed approach to understanding local interactions.

In the aggregate, our data highlight the genetic and metabolic versatility of *A. muscaria*, a globally invasive ECM fungus. While our results offer insights into the origin of introduced populations, the evolution of the *A. muscaria* lineage, and the genomic signatures of South African introductions (or their lack), these findings also emphasize many unanswered questions: how does *A. muscaria* interact with local antagonists in its novel ranges, and does enemy release or do novel weapons play a role in the spread of the fungus? Do population‐specific MFs contribute to its success? Our results offer insights into the global distribution of *A. muscaria*, creating a strong foundation for future research exploring how fungal invasions and co‐invasions shape ecosystems across the globe.

## Competing interests

None declared.

## Author contributions

Our study stems from a unique collaboration between scientists working in South Africa and the United States, and together, we span diverse countries and career stages. Our collaboration began at a US National Science Foundation‐funded workshop (grant no.: 1953299 awarded to JDH and JSB) held at Future Africa (www.futureafrica.science) in November 2022. The workshop initially focused on introducing students to invasion biology and providing bioinformatics training, using *Amanita muscaria* s.l. genomes as a teaching tool. AP and JDH conceived the study, and it was developed fully over the following 14 months. GRN designed and drafted the manuscript, leading the bioinformatic and natural product experiments and analyses. SRF, KMTL, DLN and CKS (listed in reverse alphabetical order as equal co‐second authors) contributed substantially to analyses and writing. CB also contributed significantly to analyses and writing. GRN, NPK, JWB and AP sequenced genomes. MAH, AN and AP led the historical research. Y‐WW led kinship analyses. GRN, SCP and NPK conducted the metabolic extractions and mass spectrometry analyses. Bioactivity work was designed, executed and analyzed as follows: microbial assays by GRN, CKS and NPK; insect assays by GRN, TKW, HLN, MEMM and KLC; and nematode assays by GRN, KTR and MZ. JDH, MTD and AP contributed to data analysis, interpretation and writing, with MTD and AP leading revisions to interpretations and the manuscript.

All authors (GRN, CKS, DLN, KMTL, SRF, CB, BMA, CPB, JWB, JSB, STB, JPRMC, KLC, LRC, MPAC, CD, TAD, MAH, NPK, KK, FAL, HLN, AN, MAN, MEMM, SCP, NQP, KTR, MS, RV, JMW, Y‐WW, BDW, MJW, TKW, TAZ, MZ, JDH, MTD and AP) participated in workshop discussions, contributed to initial analyses and helped edit the final manuscript. CKS, DLN, KMTL, SRF and CB contributed equally to this work.

## Disclaimer

The New Phytologist Foundation remains neutral with regard to jurisdictional claims in maps and in any institutional affiliations.

## Supporting information


**Dataset S1** Genome summary statistics.
**Dataset S2** Full metadata for *Amanita muscaria* data retrieved from prior publications.
**Dataset S3** Kinship analyses of the sequenced genomes.

**Fig. S1** Dried tissue of each mushroom prior to chemical analyses.
**Fig. S2** Determination of optimal cutoff to generate the PKS GCF predictions.
**Fig. S3** Determination of optimal cutoff to generate the Terpene GCF predictions.
**Fig. S4** Rooted phylogenies from the fully sequenced *Amanita muscaria* genomes.
**Fig. S5** Tree compatibility comparisons between different species reconstruction methods.
**Fig. S6** A cblaster analysis showing the presence and absence of genes in the ibotenic acid cluster in *Amanita muscaria* and outgroup genomes.
**Fig. S7** Whole fungal kingdom phylogeny depicting the number of Reciprocal best‐hit BLAST hits to genes in the ibotenic acid gene cluster.
**Fig. S8** Quantity of putative muscimol in every sample.
**Fig. S9** Comparison of polyketide synthases found in *Amanita* genomes.
**Fig. S10** Full GNPS output of every molecular family, including singletons.
**Fig. S11** Bioassays of metabolite extracts against Methicillin‐resistant *Staphylococcus aureus*.
**Fig. S12** Bioassays of metabolite extracts against *Pseudomonas aeruginosa*.
**Fig. S13** Bioassays of metabolite extracts against *Candida auris*.
**Fig. S14** Nematode bioassay results after treatment with several concentrations of extracts.
**Fig. S15** Viability assays in *Aedes aegypti* and *Musca domestica*.
**Fig. S16** GO‐Term map corresponding to genes that overlapped with 5 kb sliding windows containing at least 100 SNPs and where estimates of Fst corresponded to the right 5% tail of estimates.
**Fig. S17** Phylogenetic trees constructed using a codon‐aware alignment of the iboF and iboH genes.
**Methods S1** Additional information on phylogenomics, BUSCO genes, mitogenomes, bioinformatics and bioactivity assays.
**Table S1** Summary of mushrooms used in this study.
**Table S2** Summary of heterozygosity in sequenced genomes.
Please note: Wiley is not responsible for the content or functionality of any Supporting Information supplied by the authors. Any queries (other than missing material) should be directed to the *New Phytologist* Central Office.

## Data Availability

All genomic data used in our study are either publicly available through NCBI (see the Materials and Methods section and Dataset S2) or were generated by the authors and are made public with our publication. Raw sequencing reads and genome assemblies are available through NCBI BioProject (accession no.: PRJNA1372216). The screening methods used to test bioactivities and raw results are provided in Supporting Information (Figs S11–S15). The Supporting Information also provides details on genome annotation (Methods S1), mitogenome analysis (Methods S1), split decomposition of the SNP dataset (Methods S1) and targeted genome mining for amatoxin‐encoding genes (Methods S1). Supporting Datasets include a summary of genome summary statistics (Dataset S1), the gene markers used to generate Fig. 2 (Dataset S2) and the results of our kinship analysis (Dataset S3).

## References

[nph71064-bib-0001] Altschul SF , Gish W , Miller W , Myers EW , Lipman DJ . 1990. Basic local alignment search tool. Journal of Molecular Biology 215: 403–410. 2231712 10.1016/S0022-2836(05)80360-2

[nph71064-bib-0002] Anagnostakis SL . 1987. Chestnut blight – the classical problem of an introduced pathogen. Mycologia 79: 23–37.

[nph71064-bib-0003] Bağcı C , Nuhamunada M , Goyat H , Ladanyi C , Sehnal L , Blin K , Kautsar SA , Tagirdzhanov A , Gurevich A , Mantri S *et al*. 2025. BGC Atlas: a web resource for exploring the global chemical diversity encoded in bacterial genomes. Nucleic Acids Research 53: D618–D624. 39470730 10.1093/nar/gkae953 PMC11701567

[nph71064-bib-0004] Bankevich A , Nurk S , Antipov D , Gurevich AA , Dvorkin M , Kulikov AS , Lesin VM , Nikolenko SI , Pham S , Prjibelski AD *et al*. 2012. SPAdes: a new genome assembly algorithm and its applications to single‐cell sequencing. Journal of Computational Biology 19: 455–477. 22506599 10.1089/cmb.2012.0021 PMC3342519

[nph71064-bib-0005] Berch SM , Kroeger P , Finston T . 2017. The death cap mushroom (*Amanita phalloides*) moves to a native tree in Victoria, BC. Botany 95: 435–440.

[nph71064-bib-0006] Blin K , Shaw S , Steinke K , Villebro R , Ziemert N , Lee SY , Medema MH , Weber T . 2019. antiSmash 5.0: updates to the secondary metabolite genome mining pipeline. Nucleic Acids Research 47: W81–W87. 31032519 10.1093/nar/gkz310 PMC6602434

[nph71064-bib-0007] Blossey B , Nötzold R . 1995. Evolution of increased competitive ability in invasive nonindigenous plants: a hypothesis. Journal of Ecology 83: 887.

[nph71064-bib-0008] Bok JW , Balajee SA , Marr KA , Andes D , Nielsen KF , Frisvad JC , Keller NP . 2005. LaeA, a regulator of morphogenetic fungal virulence factors. Eukaryotic Cell 4: 1574–1582. 16151250 10.1128/EC.4.9.1574-1582.2005 PMC1214197

[nph71064-bib-0009] Bolger AM , Lohse M , Usadel B . 2014. Trimmomatic: a flexible trimmer for Illumina sequence data. Bioinformatics 30: 2114–2120. 24695404 10.1093/bioinformatics/btu170 PMC4103590

[nph71064-bib-0010] Boyd IL , Freer‐Smith PH , Gilligan CA , Godfray HC . 2013. The consequence of tree pests and diseases for ecosystem services. Science 342: 1235773. 24233727 10.1126/science.1235773

[nph71064-bib-0011] Braesel J , Fricke J , Schwenk D , Hoffmeister D . 2017. Biochemical and genetic basis of orsellinic acid biosynthesis and prenylation in a stereaceous basidiomycete. Fungal Genetics and Biology 98: 12–19. 27903443 10.1016/j.fgb.2016.11.007

[nph71064-bib-0012] Brakhage AA , Liebmann B . 2005. *Aspergillus fumigatus* conidial pigment and cAMP signal transduction: significance for virulence. Medical Mycology 43: 75–82. 10.1080/13693780400028967 16110796

[nph71064-bib-0013] Brancatelli GIE , Amodeo MR , Cuevas YA , Zalba SM . 2020. Invasive pines in Argentinian grasslands: lessons from control operations. Biological Invasions 22: 473–484.

[nph71064-bib-0014] Brewer JS , Souza FM , Callaway RM , Durigan G . 2018. Impact of invasive slash pine (*Pinus elliottii*) on groundcover vegetation at home and abroad. Biological Invasions 20: 2807–2820.

[nph71064-bib-0015] Bushnell B . 2015. BBMap short‐read aligner, and other bioinformatics tools. Berkeley, CA, USA: University of California.

[nph71064-bib-0016] Callaway RM , Ridenour WM . 2004. Novel weapons: invasive success and the evolution of increased competitive ability. Frontiers in Ecology and the Environment 2: 436–443.

[nph71064-bib-0017] Camazine S . 1983. Mushroom chemical defense: food aversion learning induced by hallucinogenic toxin, muscimol. Journal of Chemical Ecology 9: 1473–1481. 24408803 10.1007/BF00988513

[nph71064-bib-0018] Capella‐Gutierrez S , Silla‐Martinez JM , Gabaldon T . 2009. trimAl: a tool for automated alignment trimming in large‐scale phylogenetic analyses. Bioinformatics 25: 1972–1973. 19505945 10.1093/bioinformatics/btp348 PMC2712344

[nph71064-bib-0019] Carboué Q , Lopez M . 2021. *Amanita muscaria*: ecology, chemistry, myths. Encyclopedia 1: 905–914.

[nph71064-bib-0020] Cleary M , Nguyen D , Marciulyniene D , Berlin A , Vasaitis R , Stenlid J . 2016. Friend or foe? Biological and ecological traits of the European ash dieback pathogen *Hymenoscyphus fraxineus* in its native environment. Scientific Reports 6: 21895. 26900083 10.1038/srep21895 PMC4761999

[nph71064-bib-0021] Danecek P , Auton A , Abecasis G , Albers CA , Banks E , DePristo MA , Handsaker RE , Lunter G , Marth GT , Sherry ST *et al*. 2011. The variant call format and VCFtools . Bioinformatics 27: 2156–2158. 21653522 10.1093/bioinformatics/btr330 PMC3137218

[nph71064-bib-0022] Desprez‐Loustau ML , Robin C , Buee M , Courtecuisse R , Garbaye J , Suffert F , Sache I , Rizzo DM . 2007. The fungal dimension of biological invasions. Trends in Ecology & Evolution 22: 472–480. 17509727 10.1016/j.tree.2007.04.005

[nph71064-bib-0023] Dickie IA , Bolstridge N , Cooper JA , Peltzer DA . 2010. Co‐invasion by *Pinus* and its mycorrhizal fungi. New Phytologist 187: 475–484. 20456067 10.1111/j.1469-8137.2010.03277.x

[nph71064-bib-0024] Dickie IA , Bufford JL , Cobb RC , Desprez‐Loustau ML , Grelet G , Hulme PE , Klironomos J , Makiola A , Nuñez MA , Pringle A *et al*. 2017. The emerging science of linked plant‐fungal invasions. New Phytologist 215: 1314–1332. 28649741 10.1111/nph.14657

[nph71064-bib-0025] Dickie IA , St John MG , Yeates GW , Morse CW , Bonner KI , Orwin K , Peltzer DA . 2014. Belowground legacies of *Pinus contorta* invasion and removal result in multiple mechanisms of invasional meltdown. AoB Plants 6: plu056. 25228312 10.1093/aobpla/plu056 PMC4240229

[nph71064-bib-0026] Doidge EM . 1950. The South African fungi and lichens. Bothalia 5: 1–1094.

[nph71064-bib-0027] Drott MT , Bastos RW , Rokas A , Ries LNA , Gabaldon T , Goldman GH , Keller NP , Greco C . 2020. Diversity of secondary metabolism in *Aspergillus nidulans* clinical isolates. mSphere 5: e00156‐20. 32269157 10.1128/mSphere.00156-20 PMC7142299

[nph71064-bib-0028] Drott MT , Lazzaro BP , Brown DL , Carbone I , Milgroom MG . 2017. Balancing selection for aflatoxin in *Aspergillus flavus* is maintained through interference competition with, and fungivory by insects. Proceedings of the Royal Society B: Biological Sciences 284: 20172408. 10.1098/rspb.2017.2408 PMC5745424 29263278

[nph71064-bib-0029] Drott MT , Park SC , Wang YW , Harrow L , Keller NP , Pringle A . 2023. Pangenomics of the death cap mushroom *Amanita phalloides*, and of Agaricales, reveals dynamic evolution of toxin genes in an invasive range. The ISME Journal 17: 1236–1246. 37221394 10.1038/s41396-023-01432-x PMC10356791

[nph71064-bib-0030] Drott MT , Rush TA , Satterlee TR , Giannone RJ , Abraham PE , Greco C , Venkatesh N , Skerker JM , Glass NL , Labbe JL *et al*. 2021. Microevolution in the pansecondary metabolome of *Aspergillus flavus* and its potential macroevolutionary implications for filamentous fungi. Proceedings of the National Academy of Sciences, USA 118: e2021683118. 10.1073/pnas.2021683118 PMC8166093 34016748

[nph71064-bib-0031] Dunk CW , Lebel T , Keane PJ . 2012. Characterisation of ectomycorrhizal formation by the exotic fungus *Amanita muscaria* with *Nothofagus cunninghamii* in Victoria, Australia. Mycorrhiza 22: 135–147. 21573836 10.1007/s00572-011-0388-9

[nph71064-bib-0032] Faulstich H , Cochet‐Meilhac M . 1976. Amatoxins in edible mushrooms. FEBS Letters 64: 73–75. 1269766 10.1016/0014-5793(76)80252-9

[nph71064-bib-0033] Feng H , Meng P , Zhang S , Chen W , Wang H , Wang C . 2023. Insights from comparative transcriptome analysis in the responses of Pb‐tolerant fungi *Curvularia tsudae* to Pb stress. Ecotoxicology and Environmental Safety 249: 114476. 38321691 10.1016/j.ecoenv.2022.114476

[nph71064-bib-0034] Fuhrer BA . 1992. Rainforest fungi of Tasmania and south‐east Australia. East Melbourne, Vic., Australia: CSIRO Australia.

[nph71064-bib-0035] Fujii I , Mori Y , Watanabe A , Kubo Y , Tsuji G , Ebizuka Y . 2000. Enzymatic synthesis of 1,3,6,8‐tetrahydroxynaphthalene solely from malonyl coenzyme A by a fungal iterative type I polyketide synthase PKS1. Biochemistry 39: 8853–8858. 10913297 10.1021/bi000644j

[nph71064-bib-0036] Gao H , Zhou L , Zhang P , Wang Y , Qian X , Liu Y , Wu G . 2023. Filamentous fungi‐derived orsellinic acid‐sesquiterpene meroterpenoids: fungal sources, chemical structures, bioactivities, and biosynthesis. Planta Medica 89: 1110–1124. 37225133 10.1055/a-2099-4932

[nph71064-bib-0037] Geml J , Laursen GA , O'Neill K , Nusbaum HC , Taylor DL . 2006. Beringian origins and cryptic speciation events in the fly agaric (*Amanita muscaria*). Molecular Ecology 15: 225–239. 16367842 10.1111/j.1365-294X.2005.02799.x

[nph71064-bib-0038] Geml J , Tulloss RE , Laursen GA , Sazanova NA , Taylor DL . 2008. Evidence for strong inter‐ and intracontinental phylogeographic structure in *Amanita muscaria*, a wind‐dispersed ectomycorrhizal basidiomycete. Molecular Phylogenetics and Evolution 48: 694–701. 18547823 10.1016/j.ympev.2008.04.029

[nph71064-bib-0039] Gilchrist CLM , Booth TJ , van Wersch B , van Grieken L , Medema MH , Chooi Y‐H . 2021. cblaster: a remote search tool for rapid identification and visualization of homologous gene clusters. Bioinformatics Advances 1: vbab016. 36700093 10.1093/bioadv/vbab016 PMC9710679

[nph71064-bib-0040] Gillett J . 1962. Pest pressure, an underestimated factor in evolution. Systematics Association Publication 4: 37–46.

[nph71064-bib-0041] Global Biodiversity Information Facility (GBIF) at GBIF.org . 2023. Occcurrence download doi: 10.15468/dl.4pqxfc

[nph71064-bib-0042] Goldman GB , Gryzenhout M . 2019. Field guide to mushrooms and other fungi in South Africa. Cape Town, South Africa: Struik Nature.

[nph71064-bib-0043] Gruber B , Unmack PJ , Berry OF , Georges A . 2018. dartr: an r package to facilitate analysis of SNP data generated from reduced representation genome sequencing. Molecular Ecology Resources 18: 691–699. 29266847 10.1111/1755-0998.12745

[nph71064-bib-0044] Hayward J , Horton TR , Nuñez MA . 2015. Ectomycorrhizal fungal communities coinvading with Pinaceae host plants in Argentina: Gringos bajo el bosque. New Phytologist 208: 497–506. 25963605 10.1111/nph.13453

[nph71064-bib-0045] He L , Diedrich J , Chu YY , Yates JR 3rd . 2015. Extracting accurate precursor information for Tandem Mass Spectra by RawConverter. Analytical Chemistry 87: 11361–11367. 26499134 10.1021/acs.analchem.5b02721 PMC4777630

[nph71064-bib-0046] Hoff KJ , Stanke M . 2013. WebAugustus – a web service for training AUGUSTUS and predicting genes in eukaryotes. Nucleic Acids Research 41: W123–W128. 23700307 10.1093/nar/gkt418 PMC3692069

[nph71064-bib-0047] Johnston GA . 2014. Muscimol as an ionotropic GABA receptor agonist. Neurochemical Research 39: 1942–1947. 24473816 10.1007/s11064-014-1245-y

[nph71064-bib-0048] Kalyaanamoorthy S , Minh BQ , Wong TKF , von Haeseler A , Jermiin LS . 2017. ModelFinder: fast model selection for accurate phylogenetic estimates. Nature Methods 14: 587–589. 28481363 10.1038/nmeth.4285 PMC5453245

[nph71064-bib-0049] Karst J , Marczak L , Jones MD , Turkington R . 2008. The mutualism‐parasitism continuum in ectomycorrhizas: a quantitative assessment using meta‐analysis. Ecology 89: 1032–1042. 18481528 10.1890/07-0823.1

[nph71064-bib-0050] Katoh K , Standley DM . 2013. MAFFT multiple sequence alignment software version 7: improvements in performance and usability. Molecular Biology and Evolution 30: 772–780. 23329690 10.1093/molbev/mst010 PMC3603318

[nph71064-bib-0051] Keller NP . 2019. Fungal secondary metabolism: regulation, function and drug discovery. Nature Reviews Microbiology 17: 167–180. 30531948 10.1038/s41579-018-0121-1 PMC6381595

[nph71064-bib-0052] Kohler A , Kuo A , Nagy LG , Morin E , Barry KW , Buscot F , Canback B , Choi C , Cichocki N , Clum A *et al*. 2015. Convergent losses of decay mechanisms and rapid turnover of symbiosis genes in mycorrhizal mutualists. Nature Genetics 47: 410–415. 25706625 10.1038/ng.3223

[nph71064-bib-0053] Korunes KL , Samuk K . 2021. pixy: unbiased estimation of nucleotide diversity and divergence in the presence of missing data. Molecular Ecology Resources 21: 1359–1368. 33453139 10.1111/1755-0998.13326 PMC8044049

[nph71064-bib-0054] Lackner G , Bohnert M , Wick J , Hoffmeister D . 2013. Assembly of melleolide antibiotics involves a polyketide synthase with cross‐coupling activity. Chemistry & Biology 20: 1101–1106. 23993460 10.1016/j.chembiol.2013.07.009

[nph71064-bib-0055] Lackner G , Misiek M , Braesel J , Hoffmeister D . 2012. Genome mining reveals the evolutionary origin and biosynthetic potential of basidiomycete polyketide synthases. Fungal Genetics and Biology 49: 996–1003. 23078836 10.1016/j.fgb.2012.09.009

[nph71064-bib-0056] Le Maitre DC , van Wilgen BW , Gelderblom CM , Bailey C , Chapman RA , Nel JA . 2002. Invasive alien trees and water resources in South Africa: case studies of the costs and benefits of management. Forest Ecology and Management 160: 143–159.

[nph71064-bib-0057] Lebel T , May TW , Cooper JA , Catcheside D , Catcheside P , Haska J . 2024. Confirming the presence of five exotic species of *Amanita* in Australia and New Zealand. Swainsona 38: 1–44.

[nph71064-bib-0058] Li H . 2012. seqtk: *toolkit for processing sequences in FASTA/Q formats* . Github 767: 69.

[nph71064-bib-0059] Li H , Durbin R . 2009. Fast and accurate short read alignment with Burrows‐Wheeler transform. Bioinformatics 25: 1754–1760. 19451168 10.1093/bioinformatics/btp324 PMC2705234

[nph71064-bib-0060] Liu H , Stiling P . 2006. Testing the enemy release hypothesis: a review and meta‐analysis. Biological Invasions 8: 1535–1545.

[nph71064-bib-0061] Lumpert M , Kreft S . 2016. Catching flies with *Amanita muscaria*: traditional recipes from Slovenia and their efficacy in the extraction of ibotenic acid. Journal of Ethnopharmacology 187: 1–8. 27063872 10.1016/j.jep.2016.04.009

[nph71064-bib-0062] Manichaikul A , Mychaleckyj JC , Rich SS , Daly K , Sale M , Chen WM . 2010. Robust relationship inference in genome‐wide association studies. Bioinformatics 26: 2867–2873. 20926424 10.1093/bioinformatics/btq559 PMC3025716

[nph71064-bib-0063] Manni M , Berkeley MR , Seppey M , Simao FA , Zdobnov EM . 2021. BUSCO update: novel and streamlined workflows along with broader and deeper phylogenetic coverage for scoring of eukaryotic, prokaryotic, and viral genomes. Molecular Biology and Evolution 38: 4647–4654. 34320186 10.1093/molbev/msab199 PMC8476166

[nph71064-bib-0064] Márquez Parraguez CK . 2024. Ensayo de inducción de la simbiosis micorrícica entre el hongo exótico invasor Amanita muscaria y arboles nativos del género Nothofagus . Undergraduate thesis, Universidad de Concepción.

[nph71064-bib-0065] McGinnis S , Madden TL . 2004. BLAST: at the core of a powerful and diverse set of sequence analysis tools. Nucleic Acids Research 32: W20–W25. 15215342 10.1093/nar/gkh435 PMC441573

[nph71064-bib-0066] McKenna A , Hanna M , Banks E , Sivachenko A , Cibulskis K , Kernytsky A , Garimella K , Altshuler D , Gabriel S , Daly M *et al*. 2010. The Genome Analysis Toolkit: a MapReduce framework for analyzing next‐generation DNA sequencing data. Genome Research 20: 1297–1303. 20644199 10.1101/gr.107524.110 PMC2928508

[nph71064-bib-0067] Michelot D , Melendez‐Howell LM . 2003. *Amanita muscaria*: chemistry, biology, toxicology, and ethnomycology. Mycological Research 107: 131–146. 12747324 10.1017/s0953756203007305

[nph71064-bib-0068] Mikola P . 1970. Mycorrhizal inoculation in afforestation. International Review of Forestry Research 3: 123–196.

[nph71064-bib-0069] Milani T , Hoeksema JD , Jobbágy EG , Rojas JA , Vilgalys R , Teste FP . 2022. Co‐invading ectomycorrhizal fungal succession in pine‐invaded mountain grasslands. Fungal Ecology 60: 101176.

[nph71064-bib-0070] Miller OK , Jenkins DT . 1978. A taxonomic and nomenclatural study of the genus *Amanita* section *Amanita* for North America. Mycologia 70: 474.

[nph71064-bib-0071] Minh BQ , Schmidt HA , Chernomor O , Schrempf D , Woodhams MD , von Haeseler A , Lanfear R . 2020. Iq‐tree 2: new models and efficient methods for phylogenetic inference in the genomic era. Molecular Biology and Evolution 37: 1530–1534. 32011700 10.1093/molbev/msaa015 PMC7182206

[nph71064-bib-0072] Mudbhari S , Lofgren L , Appidi MR , Vilgalys R , Hettich RL , Abraham PE . 2024. Decoding the chemical language of *Suillus* fungi: genome mining and untargeted metabolomics uncover terpene chemical diversity. mSystems 9: e0122523. 38470040 10.1128/msystems.01225-23 PMC11019867

[nph71064-bib-0073] Navarro‐Munoz JC , Selem‐Mojica N , Mullowney MW , Kautsar SA , Tryon JH , Parkinson EI , De Los Santos ELC , Yeong M , Cruz‐Morales P , Abubucker S *et al*. 2020. A computational framework to explore large‐scale biosynthetic diversity. Nature Chemical Biology 16: 60–68. 31768033 10.1038/s41589-019-0400-9 PMC6917865

[nph71064-bib-0074] Neville P , Poumarat S . 2004. Amaniteae. Amanita, Limacella & Torrendia fungi Europaei 9. Alassio, Italy: Candusso Edizioni.

[nph71064-bib-0075] Nickles GR , Oestereicher B , Keller NP , Drott MT . 2023. Mining for a new class of fungal natural products: the evolution, diversity, and distribution of isocyanide synthase biosynthetic gene clusters. Nucleic Acids Research 51: 7220–7235. 37427794 10.1093/nar/gkad573 PMC10415135

[nph71064-bib-0076] Nuñez MA , Chiuffo MC , Torres A , Paul T , Dimarco RD , Raal P , Policelli N , Moyano J , García RA , van Wilgen BW *et al*. 2017. Ecology and management of invasive Pinaceae around the world: progress and challenges. Biological Invasions 19: 3099–3120.

[nph71064-bib-0077] Nuñez MA , Horton TR , Simberloff D . 2009. Lack of belowground mutualisms hinders Pinaceae invasions. Ecology 90: 2352–2359. 19769113 10.1890/08-2139.1

[nph71064-bib-0078] Obermaier S , Müller M . 2020. Ibotenic acid biosynthesis in the fly agaric is initiated by glutamate hydroxylation. Angewandte Chemie International Edition 59: 12432–12435. 32233056 10.1002/anie.202001870 PMC7383597

[nph71064-bib-0079] Oda T , Tanaka C , Tsuda M . 2004. Molecular phylogeny and biogeography of the widely distributed *Amanita* species, *A. Muscaria* and *A. Pantherina* . Mycological Research 108: 885–896. 15449593 10.1017/s0953756204000620

[nph71064-bib-0080] Peres MTLP , Mapeli A , Faccenda O , Gomes AT , Honda NK . 2009. Allelopathic potential of orsellinic acid derivatives. Brazilian Archives of Biology and Technology 52: 1019–1026.

[nph71064-bib-0081] Pildain MB , Marchelli P , Azpilicueta MM , Starik C , Barroetavena C . 2021. Understanding introduction history: genetic structure and diversity of the edible ectomycorrhizal fungus, *Suillus luteus*, in Patagonia (Argentina). Mycologia 113: 715–724. 34106819 10.1080/00275514.2021.1909449

[nph71064-bib-0082] Policelli N , Bruns TD , Vilgalys R , Nunez MA . 2019. Suilloid fungi as global drivers of pine invasions. New Phytologist 222: 714–725. 30586169 10.1111/nph.15660

[nph71064-bib-0083] Policelli N , Hoeksema JD , Moyano J , Vilgalys R , Vivelo S , Bhatnagar JM . 2023. Global pine tree invasions are linked to invasive root symbionts. New Phytologist 237: 16–21. 36221214 10.1111/nph.18527

[nph71064-bib-0084] Policelli N , Nuñez MA . 2025. Invasive ectomycorrhizal fungi: belowground insights from South America. New Phytologist 248: 2714–2721. 41017211 10.1111/nph.70608

[nph71064-bib-0085] Pringle A , Adams RI , Cross HB , Bruns TD . 2009. The ectomycorrhizal fungus *Amanita phalloides* was introduced and is expanding its range on the west coast of North America. Molecular Ecology 18: 817–833. 19207260 10.1111/j.1365-294X.2008.04030.x

[nph71064-bib-0086] Pringle A , Vellinga EC . 2006. Last chance to know? Using literature to explore the biogeography and invasion biology of the death cap mushroom *Amanita phalloides* (Vaill. ex Fr.:Fr.) Link. Biological Invasions 8: 1131–1144.

[nph71064-bib-0087] Prjibelski A , Antipov D , Meleshko D , Lapidus A , Korobeynikov A . 2020. Using SPAdes *de novo* assembler. Current Protocols in Bioinformatics 70: e102. 32559359 10.1002/cpbi.102

[nph71064-bib-0088] Reid DA , Eicker A . 1991. South African fungi: the genus *Amanita* . Mycological Research 95: 80–95.

[nph71064-bib-0089] Richardson DM , Higgins SI . 1998. Pines as invaders in the southern hemisphere. In: Richardson DM , ed. Ecology and biogeography of Pinus. Cambridge, UK: Cambridge University Press.

[nph71064-bib-0090] Robey MT , Caesar LK , Drott MT , Keller NP , Kelleher NL . 2021. An interpreted atlas of biosynthetic gene clusters from 1,000 fungal genomes. Proceedings of the National Academy of Sciences of the United States of America 118: e2020230118. 33941694 10.1073/pnas.2020230118 PMC8126772

[nph71064-bib-0091] Rossman AY . 2009. The impact of invasive fungi on agricultural ecosystems in the United States. In: Langor DW , Sweeney J , eds. Ecological impacts of non‐native invertebrates and fungi on terrestrial ecosystems. Dordrecht, the Netherlands: Springer Netherlands, 97–107.

[nph71064-bib-0092] Rouget M , Richardson DM , Milton SJ , Polakow D . 2001. Predicting invasion dynamics of four alien *Pinus* species in a highly fragmented semi‐arid shrubland in South Africa. Plant Ecology 152: 79–92.

[nph71064-bib-0093] Sapsford SJ , Wakelin A , Peltzer DA , Dickie IA . 2022. Pine invasion drives loss of soil fungal diversity. Biological Invasions 24: 401–414.

[nph71064-bib-0094] Schaffer AA , Aravind L , Madden TL , Shavirin S , Spouge JL , Wolf YI , Koonin EV , Altschul SF . 2001. Improving the accuracy of PSI‐BLAST protein database searches with composition‐based statistics and other refinements. Nucleic Acids Research 29: 2994–3005. 11452024 10.1093/nar/29.14.2994 PMC55814

[nph71064-bib-0095] Schroeckh V , Scherlach K , Nutzmann HW , Shelest E , Schmidt‐Heck W , Schuemann J , Martin K , Hertweck C , Brakhage AA . 2009. Intimate bacterial‐fungal interaction triggers biosynthesis of archetypal polyketides in *Aspergillus nidulans* . Proceedings of the National Academy of Sciences, USA 106: 14558–14563. 10.1073/pnas.0901870106 PMC2732885 19666480

[nph71064-bib-0096] Schüffler A . 2018. Secondary metabolites of basidiomycetes. In: Anke T , Weber D , eds. Physiology and genetics: selected basic and applied aspects. New York, NY, USA: Springer, 231–275.

[nph71064-bib-0124] Schwartz MW , Hoeksema JD , Gehring CA , Johnson NC , Klironomos JN , Abbott LK , Pringle A . 2006. The promise and the potential consequences of the global transport of mycorrhizal fungal inoculum. Ecology Letters 9: 601–616. 10.1111/j.1461-0248.2006.00910.x 16643296

[nph71064-bib-0097] Shannon P , Markiel A , Ozier O , Baliga NS , Wang JT , Ramage D , Amin N , Schwikowski B , Ideker T . 2003. Cytoscape: a software environment for integrated models of biomolecular interaction networks. Genome Research 13: 2498–2504. 14597658 10.1101/gr.1239303 PMC403769

[nph71064-bib-0098] Simao FA , Waterhouse RM , Ioannidis P , Kriventseva EV , Zdobnov EM . 2015. Busco: assessing genome assembly and annotation completeness with single‐copy orthologs. Bioinformatics 31: 3210–3212. 26059717 10.1093/bioinformatics/btv351

[nph71064-bib-0099] Simberloff D , Nuñez MA , Ledgard NJ , Pauchard A , Richardson DM , Sarasola M , Van Wilgen BW , Zalba SM , Zenni RD , Bustamante R *et al*. 2010. Spread and impact of introduced conifers in South America: lessons from other southern hemisphere regions. Austral Ecology 35: 489–504.

[nph71064-bib-0100] Smith SE , Read DJ . 2008. Mycorrhizal symbiosis. London, UK: Academic Press.

[nph71064-bib-0101] Staerck C , Vandeputte P , Gastebois A , Calenda A , Giraud S , Papon N , Bouchara JP , Fleury MJJ . 2018. Enzymatic mechanisms involved in evasion of fungi to the oxidative stress: focus on *Scedosporium apiospermum* . Mycopathologia 183: 227–239. 28639066 10.1007/s11046-017-0160-6

[nph71064-bib-0102] Stanke M , Diekhans M , Baertsch R , Haussler D . 2008. Using native and syntenically mapped cDNA alignments to improve *de novo* gene finding. Bioinformatics 24: 637–644. 18218656 10.1093/bioinformatics/btn013

[nph71064-bib-0103] Størmer FC , Janak K , Koller GEB . 2004. Ibotenic acid in *Amanita muscaria* spores and caps. Mycologist 18: 114–117.

[nph71064-bib-0104] Su YT , Liu J , Yang DN , Cai Q , Yang ZL , Chen ZH . 2023. Determination of ibotenic acid and muscimol in species of the genus *Amanita* section *Amanita* from China. Toxicon 233: 107257. 37611670 10.1016/j.toxicon.2023.107257

[nph71064-bib-0105] Tang MC , Fischer CR , Chari JV , Tan D , Suresh S , Chu A , Miranda M , Smith J , Zhang Z , Garg NK *et al*. 2019. Thioesterase‐catalyzed aminoacylation and thiolation of polyketides in fungi. Journal of the American Chemical Society 141: 8198–8206. 31051070 10.1021/jacs.9b01083 PMC6589186

[nph71064-bib-0106] Tannous J , Cosetta CM , Drott MT , Rush TA , Abraham PE , Giannone RJ , Keller NP , Wolfe BE . 2023. LaeA ‐regulated fungal traits mediate bacterial community assembly. mBio 14: e0076923. 10.1128/mbio.00769-23 PMC10294623 37162223

[nph71064-bib-0107] Torchin ME , Lafferty KD , Dobson AP , McKenzie VJ , Kuris AM . 2003. Introduced species and their missing parasites. Nature 421: 628–630. 12571595 10.1038/nature01346

[nph71064-bib-0108] Treseder KK , Lennon JT . 2015. Fungal traits that drive ecosystem dynamics on land. Microbiology and Molecular Biology Reviews 79: 243–262. 25971588 10.1128/MMBR.00001-15 PMC4429240

[nph71064-bib-0109] Tulloss R , Yang ZL . 2010. Amanitaceae studies. [WWW document] URL https://www.amanitaceae.org.

[nph71064-bib-0110] Tulloss RE , Rodríguez‐Caycedo C , Hughes KW , Geml J , Kudzma LV , Wolf BE , Arora D . 2015. Nomenclatural changes in *Amanita* II. Amanitaceae 1: 1–6.

[nph71064-bib-0111] Tuno N , Takahashi KH , Yamashita H , Osawa N , Tanaka C . 2007. Tolerance of Drosophila flies to ibotenic acid poisons in mushrooms. Journal of Chemical Ecology 33: 311–317. 17195114 10.1007/s10886-006-9228-3

[nph71064-bib-0112] Vargas N , Goncalves SC , Franco‐Molano AE , Restrepo S , Pringle A . 2019. In Colombia the Eurasian fungus *Amanita muscaria* is expanding its range into native, tropical *Quercus humboldtii* forests. Mycologia 111: 758–771. 31408397 10.1080/00275514.2019.1636608

[nph71064-bib-0113] Veerabahu A , Banik MT , Lindner DL , Pringle A , Jusino MA . 2025. Invasive golden oyster mushrooms are disrupting native fungal communities as they spread throughout North America. Current Biology 35: 3994–4002. 40675152 10.1016/j.cub.2025.06.049

[nph71064-bib-0114] Vellinga EC , Wolfe BE , Pringle A . 2009. Global patterns of ectomycorrhizal introductions. New Phytologist 181: 960–973. 19170899 10.1111/j.1469-8137.2008.02728.x

[nph71064-bib-0115] Walton J . 2018. The cyclic peptide toxins of *Amanita* and other poisonous mushrooms. Cham, Switzerland: Springer International.

[nph71064-bib-0116] Wang M , Carver JJ , Phelan VV , Sanchez LM , Garg N , Peng Y , Nguyen DD , Watrous J , Kapono CA , Luzzatto‐Knaan T *et al*. 2016. Sharing and community curation of mass spectrometry data with Global Natural Products Social Molecular Networking. Nature Biotechnology 34: 828–837. 10.1038/nbt.3597 PMC5321674 27504778

[nph71064-bib-0117] Wang YW , McKeon MC , Elmore H , Hess J , Golan J , Gage H , Mao W , Harrow L , Goncalves SC , Hull CM *et al*. 2023. Invasive Californian death caps develop mushrooms unisexually and bisexually. Nature Communications 14: 6560. 10.1038/s41467-023-42317-z PMC10598064 37875491

[nph71064-bib-0118] Wasson RG . 1969. Soma divine mushroom of immortality. Mycologia 61: 849.

[nph71064-bib-0119] van Wilgen BW , Richardson DM . 2012. Three centuries of managing introduced conifers in South Africa: benefits, impacts, changing perceptions and conflict resolution. Journal of Environmental Management 106: 56–68. 22562012 10.1016/j.jenvman.2012.03.052

[nph71064-bib-0120] Wolfe BE , Tulloss RE , Pringle A . 2012. The irreversible loss of a decomposition pathway marks the single origin of an ectomycorrhizal symbiosis. PLoS ONE 7: e39597. 22815710 10.1371/journal.pone.0039597 PMC3399872

[nph71064-bib-0121] Won TH , Bok JW , Nadig N , Venkatesh N , Nickles G , Greco C , Lim FY , González JB , Turgeon BG , Keller NP *et al*. 2022. Copper starvation induces antimicrobial isocyanide integrated into two distinct biosynthetic pathways in fungi. Nature Communications 13: 4828. 10.1038/s41467-022-32394-x PMC9381783 35973982

[nph71064-bib-0122] Xu W , Chooi Y‐H , Choi JW , Li S , Vederas JC , Da Silva NA , Tang Y . 2013. LovG: the thioesterase required for dihydromonacolin L release and lovastatin nonaketide synthase turnover in lovastatin biosynthesis. Angewandte Chemie International Edition 52: 6472–6475. 23653178 10.1002/anie.201302406 PMC3844545

[nph71064-bib-0123] Yu GC , Smith DK , Zhu HC , Guan Y , Lam TTY . 2017. ggtree: an R package for visualization and annotation of phylogenetic trees with their covariates and other associated data. Methods in Ecology and Evolution 8: 28–36.

