# Supplementary material for: Equipped for success: genomes and metabolomes of the European Amanita muscaria are conserved in its novel South African range
Source: New Phytol. 2026 Mar 12;250(3):1863–83. doi: 10.1111/nph.71064 (PMC13062730; doi:10.1111/nph.71064)
Supplement: Supplementary file 2 — Fig. S1 Dried tissue of each mushroom prior to chemical analyses. Fig. S2 Determination of optimal cutoff to generate the PKS GCF predictions. Fig. S3 Determination of optimal cutoff to generate the Terpene GCF predictions. Fig. S4 Rooted phylogenies from the fully sequenced Amanita muscaria genomes. Fig. S5 Tree compatibility comparisons between different species reconstruction methods. Fig. S6 A cblaster analysis showing the presence and absence of genes in the ibotenic acid cluster in Amanita muscaria and outgroup genomes. Fig. S7 Whole fungal kingdom phylogeny depicting the number of Reciprocal best‐hit BLAST hits to genes in the ibotenic acid gene cluster. Fig. S8 Quantity of putative muscimol in every sample. Fig. S9 Comparison of polyketide synthases found in Amanita genomes. Fig. S10 Full GNPS output of every molecular family, including singletons. Fig. S11 Bioassays of metabolite extracts against Methicillin‐resistant Staphylococcus aureus. Fig. S12 Bioassays of metabolite extracts against Pseudomonas aeruginosa. Fig. S13 Bioassays of metabolite extracts against Candida auris. Fig. S14 Nematode bioassay results after treatment with several concentrations of extracts. Fig. S15 Viability assays in Aedes aegypti and Musca domestica. Fig. S16 GO‐Term map corresponding to genes that overlapped with 5 kb sliding windows containing at least 100 SNPs and where estimates of Fst corresponded to the right 5% tail of estimates. Fig. S17 Phylogenetic trees constructed using a codon‐aware alignment of the iboF and iboH genes. Methods S1 Additional information on phylogenomics, BUSCO genes, mitogenomes, bioinformatics and bioactivity assays. Table S1 Summary of mushrooms used in this study. Table S2 Summary of heterozygosity in sequenced genomes. Please note: Wiley is not responsible for the content or functionality of any Supporting Information supplied by the authors. Any queries (other than missing material) should be directed to the New Phytologist Central Office. [file NPH-250-1863-s002.pdf]

New Phytologist

New Phytologist Supporting Information

Article title: Equipped for success: Genomes and metabolomes of the European *Amanita muscaria* are conserved in its novel South African range

Authors:

First: Grant R. Nickles[1]

Second:

Cecelia K. Stokes[2,3], Deborah L. Narh[4,5], Kira M.T. Lynn[4], Savannah R. Fuqua[6], Corbin Bryan[2]

Middle:

Brooke M. Allen[7], Christopher P. Bivins[8], Jin Woo Bok[1], J. Stephen Brewer[7], Sikelela T. Buthelezi[9], Jahiya P.R.M. Clark[10], Kerri L. Coon[11], Lauren R. Corby[12], Martin P.A. Coetzee[4], Claudette Dewing[4], Tuan A. Duong[4], Mathew A. Harris[9,13], Nancy P. Keller[1,14], Katlego Kopotsa[9,15], Frances A. Lane[4], Holly L. Nichols[11,16], Alandie Nieuwoudt[9], Martin A. Nuñez[17], Miguel E. Medina Munoz[11], Sung Chul Park[1], Nam Q. Pham[4], Kaetlyn T. Ryan[18], Myriam Solís[4], Rytas Vilgalys[19], Jasmine Marea Wallace[20], Yen-Wen Wang[21], Brenda D. Wingfield[4], Michael J. Wingfield[9], Travis K. Worley[11,16], Taylor A. Zallek[22], Mostafa Zamanian[18]

Last: Jason D. Hoeksema[7], Milton T. Drott[23], Anne Pringle[2,11]

The following Supporting Information is available for this article:

Methods S1

Split decomposition of SNP dataset and phylogenomic tree construction

Split decomposition was used to reconstruct the phylogenetic tree, and the tree was built from the filtered SNP dataset in order to identify potential errors or artifacts. The split decomposition method is particularly good at identifying potential errors or artifacts in a dataset, including conflicting signals, long-branch attraction, or incomplete lineage sorting. The Variant Call Format (VCF) files were initially converted into the nexus format using the vcf2phylip program (v2.0) (Ortiz, 2019) . Subsequently, the SplitsTree 4 program (Huson, 1998) was used to align the dataset (933,863 bp alignment) and to generate a neighbor network. The inference algorithm "gradient projection" was employed, and the circling order was set as SplitsTree4 on (Huson & Bryant, 2006) .

Due to the computational challenges associated with the size of the SNP dataset, which contained over 900,000+ sites, generating a 1,000 bootstrap maximum likelihood tree was not feasible. Instead, we constructed a 100-bootstrap replicate-supported tree using the SplitsTree 4 program (Huson, 1998; Huson & Bryant, 2006) .

Detecting BUSCO genes to check genome annotation quality

BUSCO (v3.0.2) was run on each genome using the agaricales_odb10 database (v: 2020-08-05) (Simão et al. 2015). Complete single copy orthologous genes (1,921) were identified and extracted from the BUSCO analyses. Genes with less than 25 orthologs across all genomes were ignored for all subsequent analysis, yielding a final count of 1,465 BUSCO genes.

Mitogenomes assembly, annotation, and phylogenetics analyses

Mitogenomes were *de novo* assembled from clean reads using NOVOPlasty v 4.3 (Dierckxsens et al. 2017). Annotation of the mitogenomes was performed using GeSeq (https://chlorobox.mpimp-golm.mpg.de/geseq.htmL) (Tillich *et al.*, 2017) . All mitogenomes were aligned using MAFFT v. 7 (http://mafft.cbrc.jp/alignment/server/) (Katoh & Standley, 2013) , then confirmed visually in MEGA v. 7 as necessary. The Maximum Likelihood (ML) analyses were conducted using RaxML v. 8.2.4 (Stamatakis, 2014) on the CIPRES Science Gateway v. 3.3 (Towns *et al.*, 2011) with a default GTR substitution matrix and 1,000 rapid bootstrap replicates. The phylogenetic tree was viewed and edited in MEGA v. 7 (Kumar *et al.*, 2018) .

Bioinformatic screening for amatoxin-encoding and associated genes

Recent research has raised questions about the production of amatoxins in *A. muscaria*. Amatoxins are part of a larger gene family termed 'MSDINs' after the Met-Ser-Asp-Ile-Asn protein motif commonly found in these genes. MSDIN genes encode propeptides subsequently processed by a prolyl oligopeptidase 'POPB', a protein diverged from the housekeeping protein 'POPA'; processing results in a mature cyclized product. To clarify the amatoxin-producing potential of *A. muscaria*, we scanned genomes using the MSDIN-finding pipeline developed by (Drott *et al.*, 2023) using default parameters. While this pipeline is effective across diverse Agaricales species, to avoid the possibility that *A. muscaria* MSDINs do not conform to default parameters (e.g., have a non-canonical intron length), we scanned the results of the initial BLAST screening in all six reading frames for the three of the most infamous amatoxins: alpha amanitin (IWGIGCNP), beta amanitin (IWGIGCDP), phallacidin (AWLVDCP), and phalloidin (AWLATCP). Additionally, using methods and query datasets identical to (Drott *et al.*, 2023), we identified any sequences corresponding to POPA and POPB using tBLASTn. A large window around each hit was annotated using Augustus and the three best hits from each protein (up to six hits total) were aligned using MAFFT (Katoh *et al.*, 2002), trimmed using trimal (Capella-Gutiérrez *et al.*, 2009), and used to construct a phylogeny in IQTREE2 (Minh *et al.*, 2020). Resulting phylogenies were manually inspected to determine if any predicted proteins fell within known POPA or POPB clades.

Antimicrobial disc assay

We ran a standard disc diffusion assay against three clinically relevant human pathogens available to us at UW-Madison: Methicillin-resistant *Staphylococcus aureus* (MRSA; Gram-positive), *Pseudomonas aeruginosa* (Gram-negative), and *Candida auris* (a Saccharomycetes yeast). *C. auris* was grown on YPD media and *P. aeruginosa* and *S. aureus* were grown on LB + agar. Extracts were prepared at high concentrations of 2.5 mg/mL and 5 mg/mL, and 20 µL of each extract was applied to the paper assay discs. We included 20 µL of pure MeOH in addition to a blank paper as negative controls in the assay. Plates were grown overnight and imaged the following day.

Nematode bioactivity screening

Nematodes were prepared depending on species and assay phenotype as follows: *C. elegans:* N2 (Bristol) strain worms were maintained on NGM plates seeded with *E. coli* OP50 at 20°C. Approximately 18 hours prior to assay set up, gravid worms were synchronized via standard bleaching procedures, and embryos were hatched overnight in filter sterilized K media. Titering of larvae, preparation of food mixture, and plate

incubation was performed as previously described (Wheeler *et al.*, 2023). For development assays, solutions were aliquoted into empty 96-well plates (Greiner Bio-One 655180) along with DMSO negative controls and 50μM albendazole sulfoxide positive controls. Next, food solution and 50 *C. elegans* L1s were added per well. For motility assays, 100 *C. elegans* L1s and double the food quantity was added to plates without extract solutions. After 48 hours, assay plates were rinsed with M9 using an AquaMax2000 plate washer (Molecular Devices). To development plates, sodium azide was added (final concentration of 200mM) to paralyze the worms. To motility plates, either extract solutions, a DMSO negative control, or a 500uM levamisole positive control was added, and plates were incubated for an additional 20 minutes. Assay plates were then imaged using an ImageXpress Nano (Molecular Devices), and images were analyzed using the wormsize_intensity_cellpose module (development plates) or motility (motility plates) modules of wrmXpress v1.4.0 (Wheeler *et al.*, 2022a). *Brugia* spp. microfilariae were obtained through the NIH/NIAID Filariasis Research Reagent Resource Center (FR3); morphological voucher specimens are stored at the Harold W. Manter Museum at the University of Nebraska, accession numbers P2021-2032 (Michalski *et al.*, 2011). Parasites were shipped and maintained in RPMI 1640 culture media with 0.1mg/mL penicillin/streptomycin at 37°C with 5% atmospheric CO_2. *Brugia pahangi* and *Brugia* malayi were used interchangeably in screening. Motility and viability assays were setup as previously described (Wheeler *et al.*, 2022b). Images were acquired on an ImageXpress Nano and analyzed using the motility and mf_celltox modules of wrmXpress (Wheeler *et al.*, 2022a). All phenotype measurements were analyzed using R software with tidyverse packages for statistical analysis.

Insect source

Aedes aegypti eggs, strain Liverpool (LVP), were kindly provided by the UW-Madison insectary, where insects are maintained at 28 °C with a 16h light: 8h dark photoperiod and 70% relative humidity (Airs *et al.*, 2019). *Musca domestica* has been reared in continuous culture in the Coon laboratory at UW-Madison since 2021.

Mass mosquito rearing for experimental setup

Larvae were mass reared in metallic pans and were fed a fine mix of rat chow (Purina): lactalbumin: torula yeast (1:1:1) (Coon *et al.*, 2016). All developmental stages were maintained at 28 °C, with a 16h light: 8h dark photoperiod in a biological incubator (Percival, model I-36VL).

Housefly rearing for experimental setup

Adult houseflies in the Coon laboratory colony are maintained at 25°C in mesh-screened cages on a dry mix of 1:1 powdered milk:sugar and water provided in a deli cup with paper towels serving as wicks.

Sterile sucrose

Sucrose (Alfa Aesar, #36508) at 10% w/v was filtered through a 0.22 µm syringe filter (Fisherbrand, #09-720-004) for sterility and 50 mL aliquots were kept at 4°C until needed.

Adult mosquito inoculation

Fungal extracts 20031-a, 11667 and Ring1 at a stock concentration of 100 mg/mL were diluted to a working concentration of 1 mg/mL in 1 mL of 10% sucrose and administered *per os* via a cotton wick. 1% DMSO in 10% sucrose was used as a control. Three cages of 20 adult *Ae. aegypti* LVP, sampled 24-48 hours after emergence from the pupal stage, were used per treatment.

Mosquito larvae inoculation

Aedes aegypti LVP larvae were challenged with 1 mg/mL of extracts 20031-a, 1167, and Ring1. Larvae were reared in 24-well plates on standard diet (1:1:1 rat chow + lactalbumin + inactive yeast) with eight replicate wells per extract alongside two controls: a food-only control and a 1% DMSO control. Five first instar larvae were placed in 500 uL of deionized water, and 20 uL of a 14 mg/mL food slurry was provided daily. First instar larvae were challenged with extracts on day one. Due to high mortality in all extract treatments, an additional cohort of larvae was challenged after 7 days of development to demonstrate differences in larvicidal effect in late-instar larvae. Larval survival was measured at 24, 48, and 72h post-challenge.

Adult housefly inoculation

For houseflies, an equal sex ratio of 20 adult *Musca domestica*, 3-4 days old, were used per treatment. During treatment, treatment groups were housed independently in half-gallon mason jars with 0.3 µM filter discs serving as lids. All jars were provisioned with approximately 2 mL each of 1:1 powdered milk:sugar in 15 mL vials as a food source. As in adult mosquito experiments, fungal extracts 20031-a, 11667 and Ring1 at a stock concentration of 100 mg/mL were diluted to a working concentration of 1 mg/mL in 1mL of 10% sucrose and administered *per os* via a cotton wick. 1% DMSO in 10% sucrose was used as a control. Dead flies were counted every 24 hours for 72 hours post-inoculation. Three independent replicates were performed per treatment.

New Phytologist

Supporting Tables

Mushroom ID	Continent	Genome	Metabolite	Antibiotic Screening	Nematicidal Screening	Insect Screening
11662	AF	Y	Y	Y	Y	n/a
11663	AF	Y	Y	Y	Y	n/a
11664	AF	Y	Y	Y	n/a	n/a
11665	AF	Y	Y	Y	n/a	n/a
11666	AF	Y	Y	Y	n/a	n/a
11667	AF	Y	Y	Y	Y	Y
11668	AF	Y	Y	Y	n/a	n/a
11669	AF	Y	Y	Y	n/a	n/a
11670	AF	Y	Y	Y	n/a	n/a
11671	AF	n/a	Y	Y	n/a	n/a
20031	NA	Y	Y	Y	Y	n/a
20031-a	NA	n/a	Y	Y	Y	Y
20045-a	NA	Y	Y	Y	Y	n/a
20045-b	NA	n/a	Y	Y	n/a	n/a
Frag1	EU	Y	Y	Y	n/a	n/a
Gril1	EU	Y	Y	Y	n/a	n/a
Kara3	EU	Y	Y	Y	Y	n/a
Nagy-Heves-A	EU	Y	Y	Y	Y	n/a
Nagy-Heves-B	EU	Y	Y	Y	n/a	n/a
Nes1	EU	Y	Y	Y	n/a	n/a
NesPan3	EU	Y	Y	Y	Y	Y
Ring1	EU	Y	Y	Y	Y	Y
Roed3	EU	Y	Y	Y	n/a	n/a
Skrap3	EU	Y	Y	Y	n/a	n/a

Sogn5	EU	Y	Y	Y	n/a	n/a
Wirz3	EU	Y	Y	Y	n/a	n/a
Aus332	AU	Y	n/a	n/a	n/a	n/a
NzAUS95	AU	Y	n/a	n/a	n/a	n/a

Table S1: Summary of mushrooms used in the study. The table specifies the continent of origin for each mushroom, whether its genome was sequenced, whether its metabolites were extracted, and whether the metabolic extract was used in bioactivity assays. AF=Africa, NA=North America, EU=Europe, AU=Australia, Y=Yes, n/a=not applicable.

Isolate	Heterozygosity	Std Dev
11662_S339	0.0674	
11663_S340	0.0613	
11664_S341	0.0661	
11665_S342	0.0620	
11666_S343	0.0724	
11667_S344	0.0723	
11668_S345	0.0706	
11669_S346	0.0704	
11670_S347	0.0733	
20031_S348	0.1003	
20045_S349	0.0971	
Aus332_S350	0.0725	
Frag1_S351	0.0849	
Gril1_S352	0.0888	
Kara3_S353	0.0833	
NagyHevesB_S355	0.0902	
NagyHevesA_S354	0.0895	
Nes1_S356	0.0833	
NesPan3_S357	0.0818	
NzAUS95_S358	0.0786	
Ring1_S359	0.0631	
Roed3_S360	0.0443	
Skrap3_S361	0.0828	
Sogn5_S362	0.0837	
Wirz_S363	0.0828	
**Average**	0.0769	0.0126
Euro Average	0.0799	0.0132
SA Average	0.0684	0.0045

Table S2: Summary of heterozygosity in sequenced genomes. Heterozygosity was determined across all variant sites

Supplementary Figures

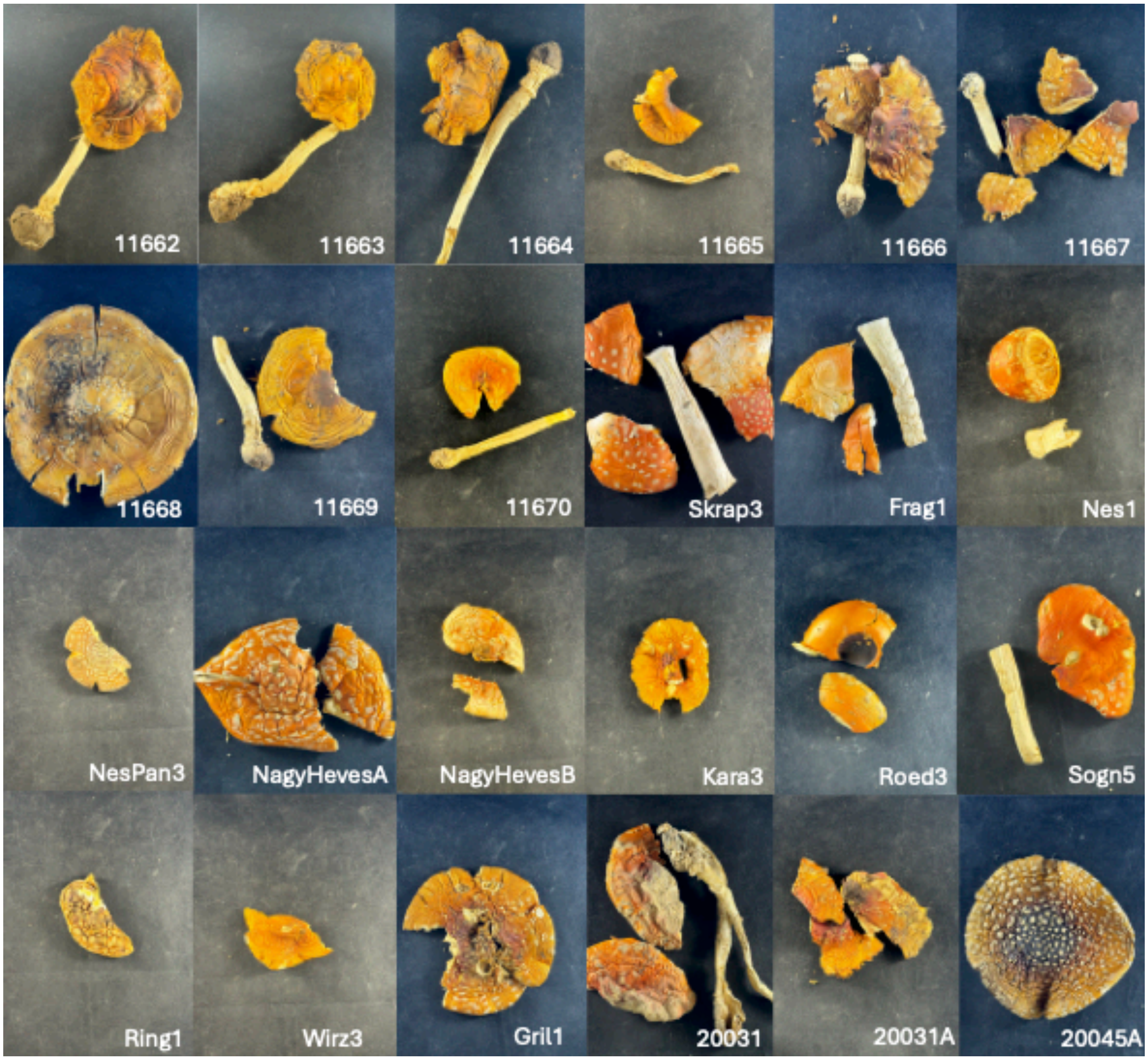

Fig. S1 Dried tissue of each mushroom prior to chemical analyses. All images taken with an iPhone prior to pulverization and metabolic extraction. We are missing photos of 11671 and 20045-b.

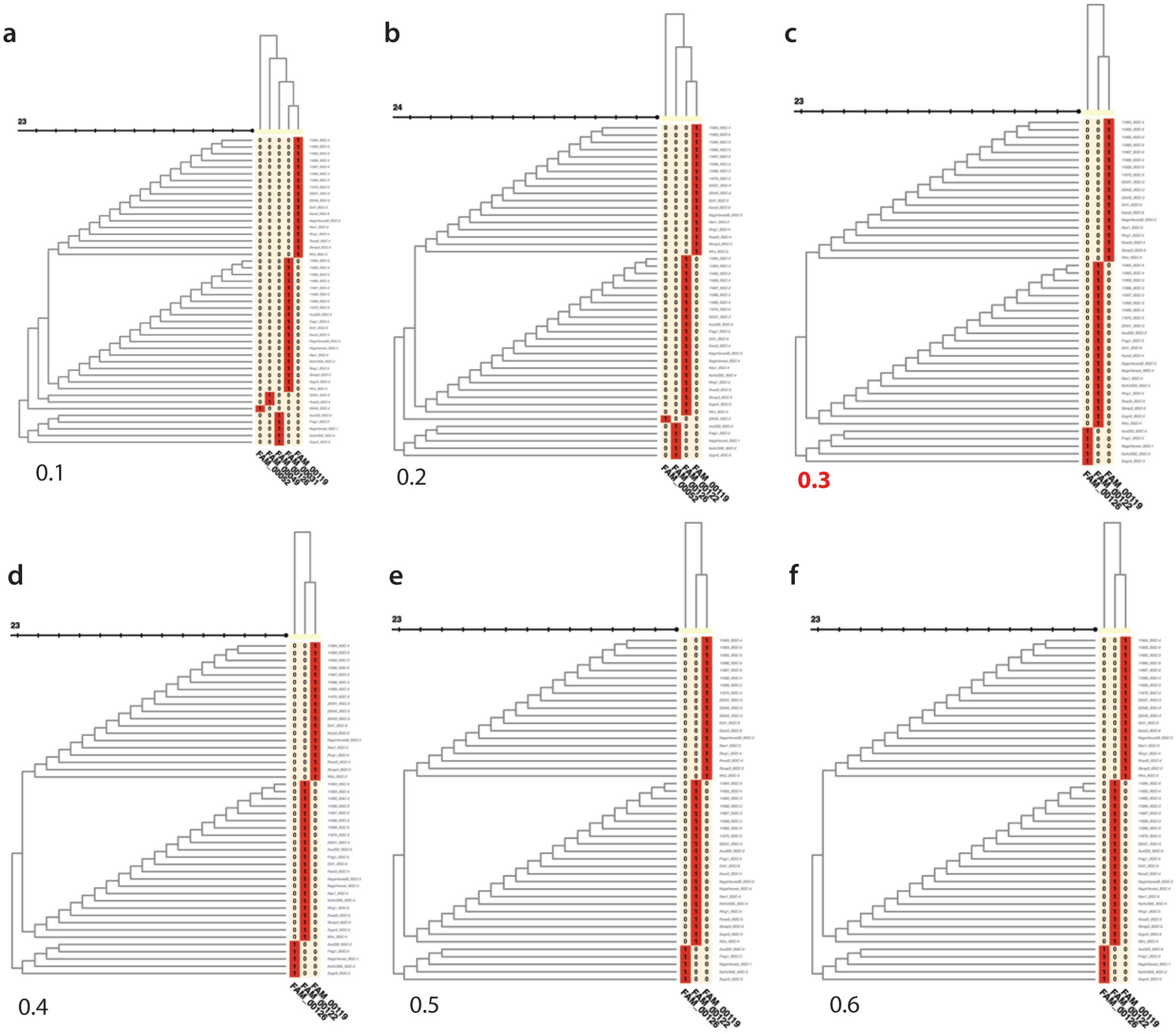

Fig. S2 Determination of optimal cutoff to generate the PKS GCF predictions
Optimization of the polyketide-synthase (PKS) gene cluster family (GCF) cutoff using the program BiG-SCAPE. Subplots (a-f) indicate absence/presence heatmaps generated by clustering the genomes based on GCF absence/presence values. The deemed optimal cutoff, 0.3, is indicated with red font.

New Phytologist

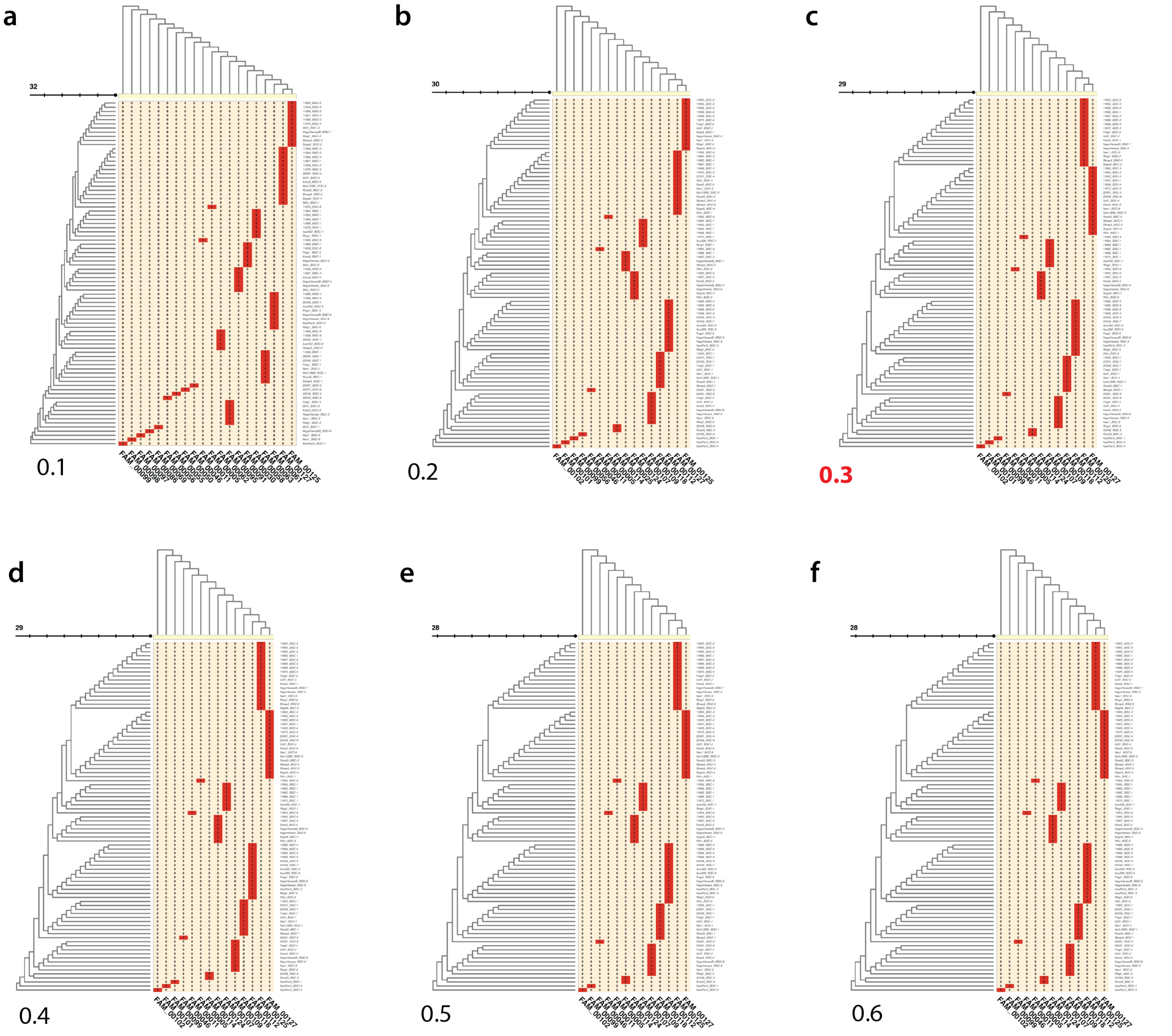

Fig. S3 Determination of optimal cutoff to generate the Terpene GCF predictions
Optimization of the terpene gene cluster family (GCF) cutoff using the program BiG-SCAPE. Subplots (a-f) indicate absence/presence heatmaps generated by clustering the genomes based on GCF absence/presence values. The deemed optimal cutoff, 0.3, is indicated with red font.

New Phytologist

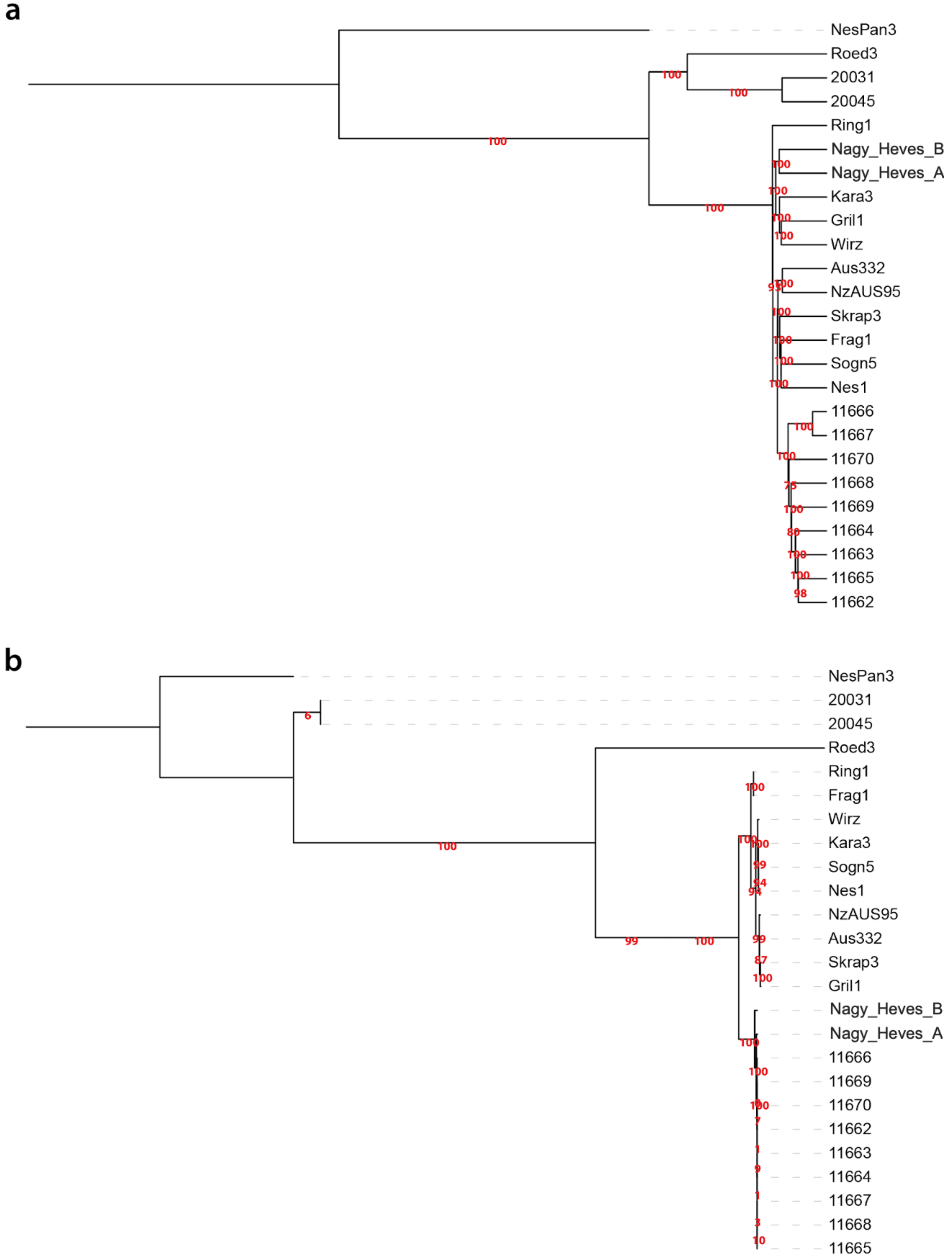

Fig. S4 Rooted phylogenies from the fully sequenced *Amanita muscaria* genomes using (a) SNP data and (b) mitochondrial genomes. Bootstrap values are displayed on branches.

New Phytologist

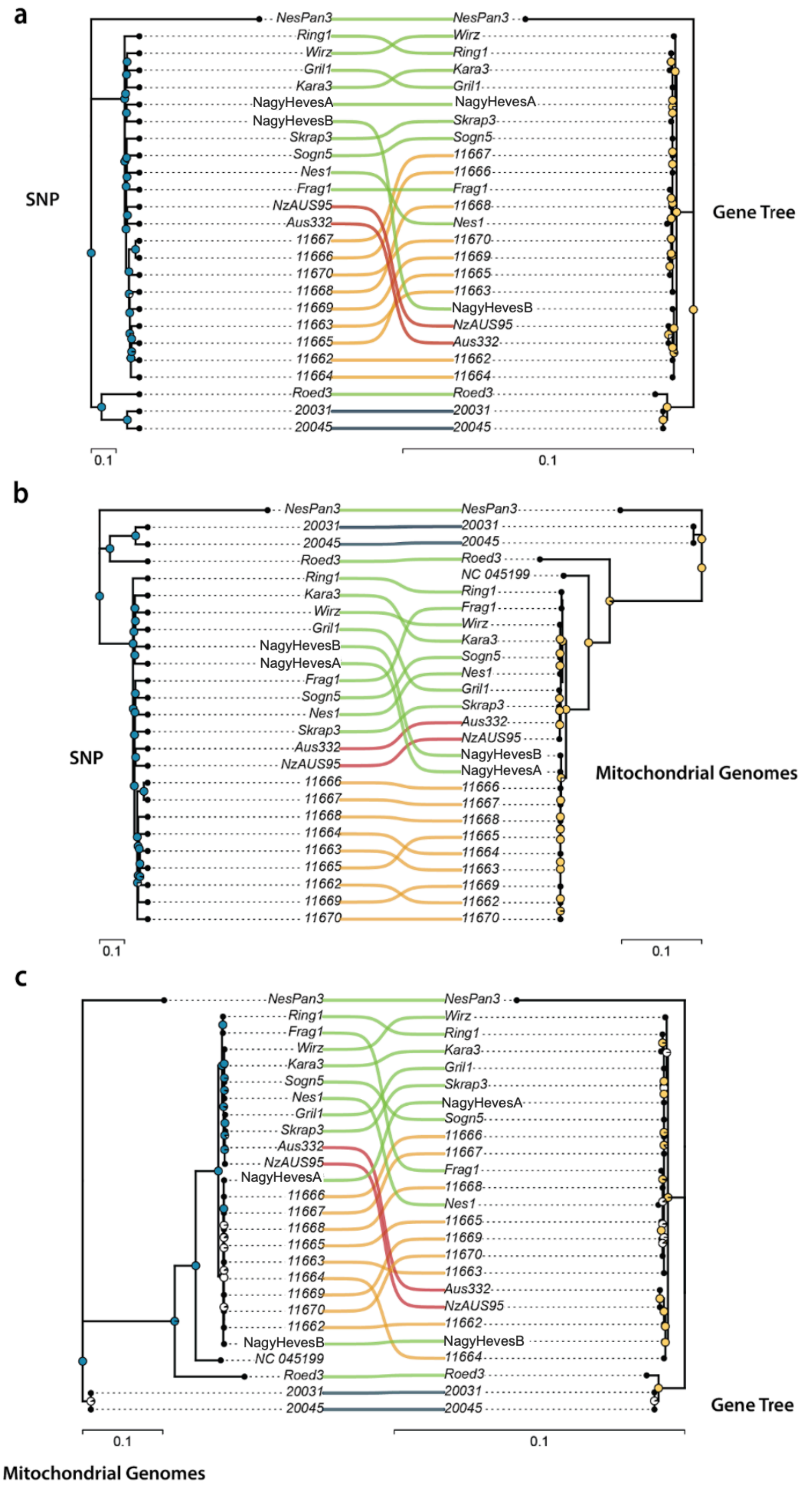

New Phytologist

Fig. S5 Tree compatibility comparisons between different species reconstruction methods. The line colors are as follows: red = Australia, green = Europe, blue = USA, yellow = South Africa. (a) SNP data tree compared to gene marker tree. (b) SNP data tree compared to mitochondrial genomes tree. (c) Mitochondrial genomes tree compared to gene marker tree.

New Phytologist

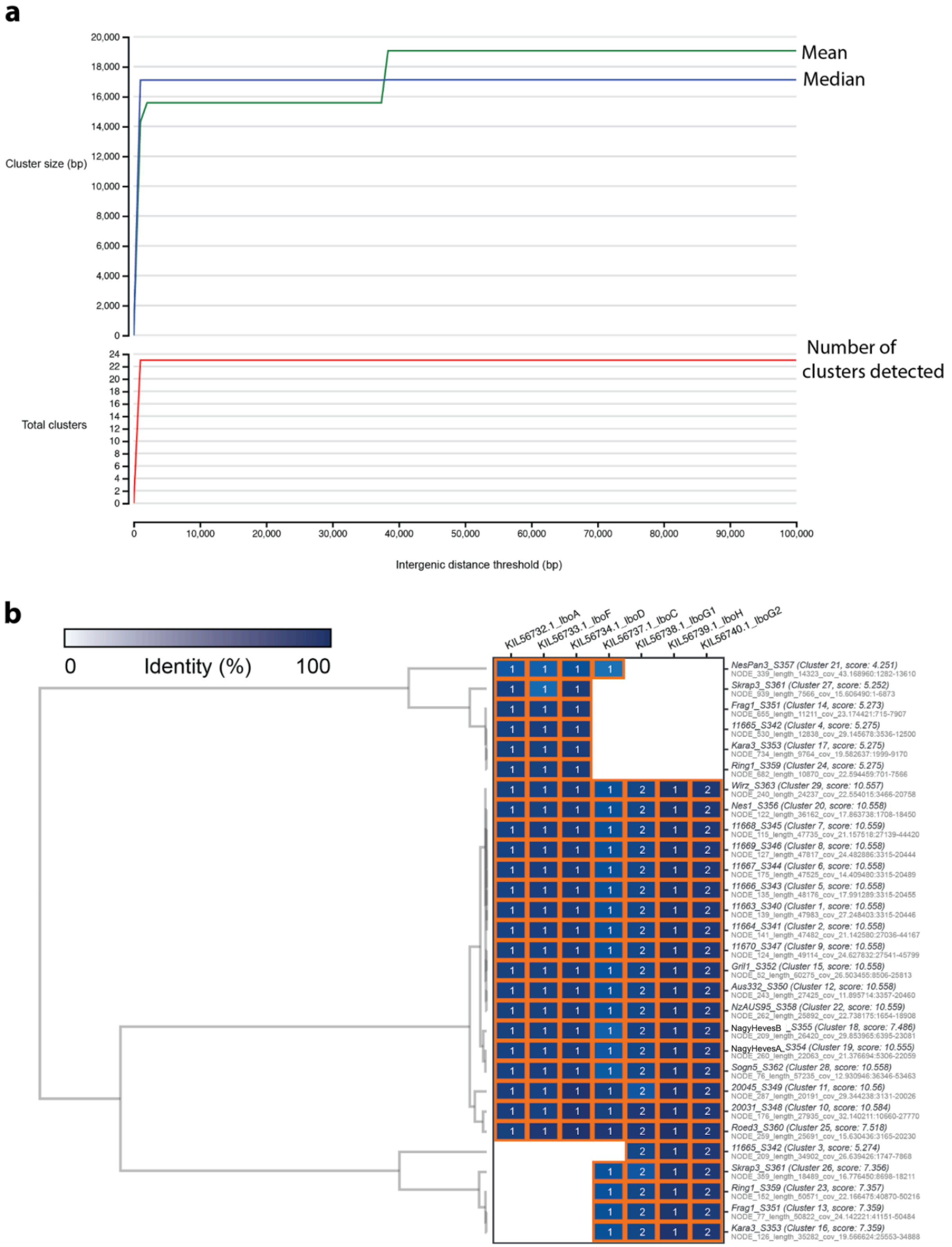

Fig. S6 A cblaster analysis showing the presence and absence of genes in the ibotenic acid cluster in the *A. muscaria* and outgroup genome.

(a) Estimated genomic neighborhood (gne) analysis to determine the optimal maximum inter-hit gap parameter for running cblaster. (**b**) Output of cblaster when using an inter-hit gap parameter of 3000 bp as determined from the gne analysis. All four occurrences of putative split clusters were manually verified to be resulting from contigs or sequencing error and are not biologically relevant. Each column is labeled with the accession number of the protein encoded by the respective gene. For each row, labels to the right indicate the genome (top) and the location of the gene cluster within the genome (bottom). The number in each box indicates the number of proteins that matched to the query sequence in the locus.

New Phytologist

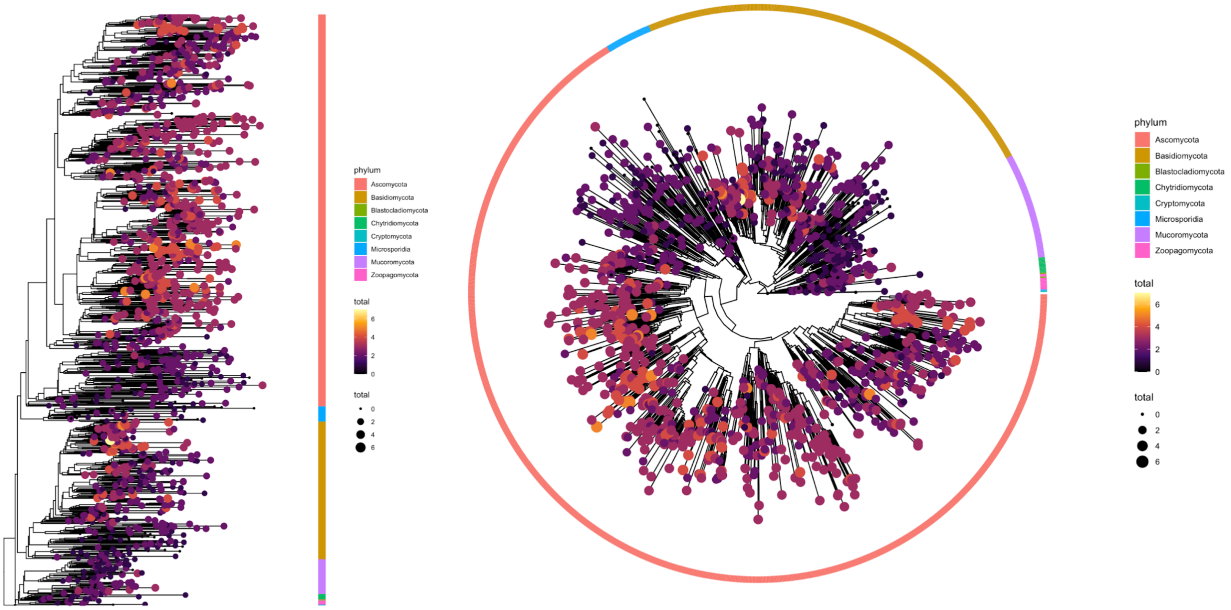

Fig. S7 Whole fungal kingdom phylogeny depicting the number of reciprocal best-hit BLAST hits to genes in the ibotenic acid gene cluster. The same tree is presented twice as some patterns are more easily visualized in the vertical tree (left) while others are clearer on the circular tree (right). While putative orthologs were found across the fungal kingdom, the most common hits were from highly diversified protein families (e.g., P450) and are more likely to result from spurious associations or ancient ancestry in putative orthologs. None of the hits in genomes not already known to produce ibotenic acid contained five or more putative orthologs clustered within 30 kb of each other. These results are consistent with the evolution of ibotenic acid from ancient gene families common in primary metabolism. While the placement of microsporidia in this phylogeny is incorrect, the grouping of species into this and other clades is correct and sufficient for depicting the patterns we address.

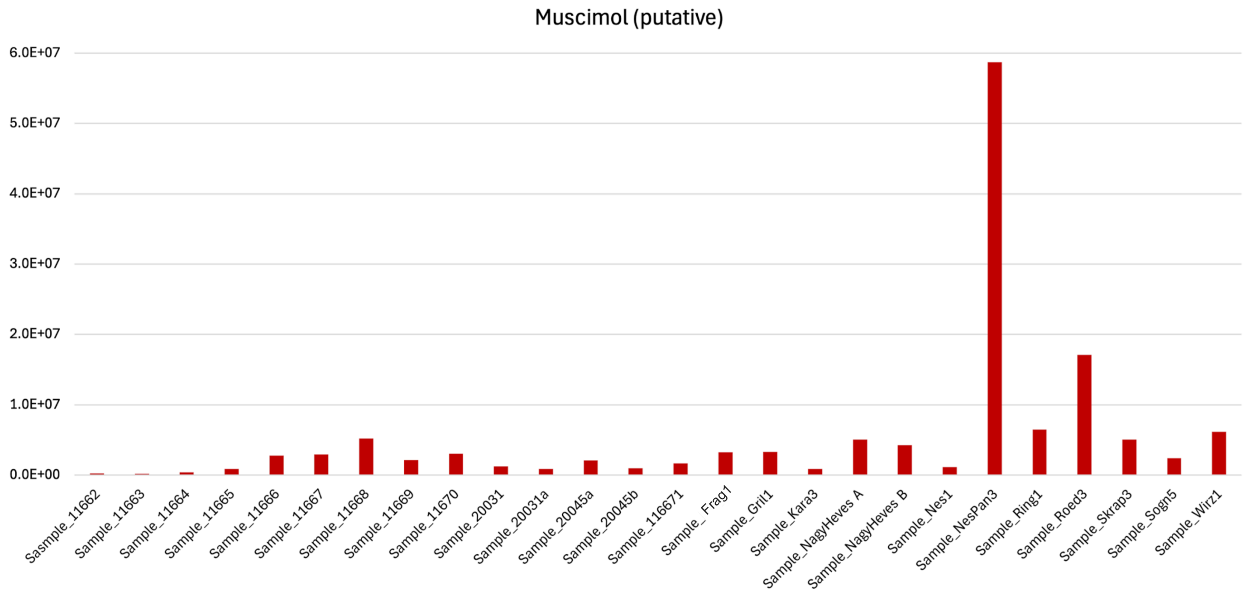

Fig. S8 Quantity of putative muscimol in every sample.
Data were generated by searching for the MS/MS fragmentation pattern of muscimol from *Amanita* extracts ([M+H]+= 115.0503, tR = 1.30). The muscimol structure was putatively confirmed *in silico* with SIRIUS software structure prediction.

New Phytologist

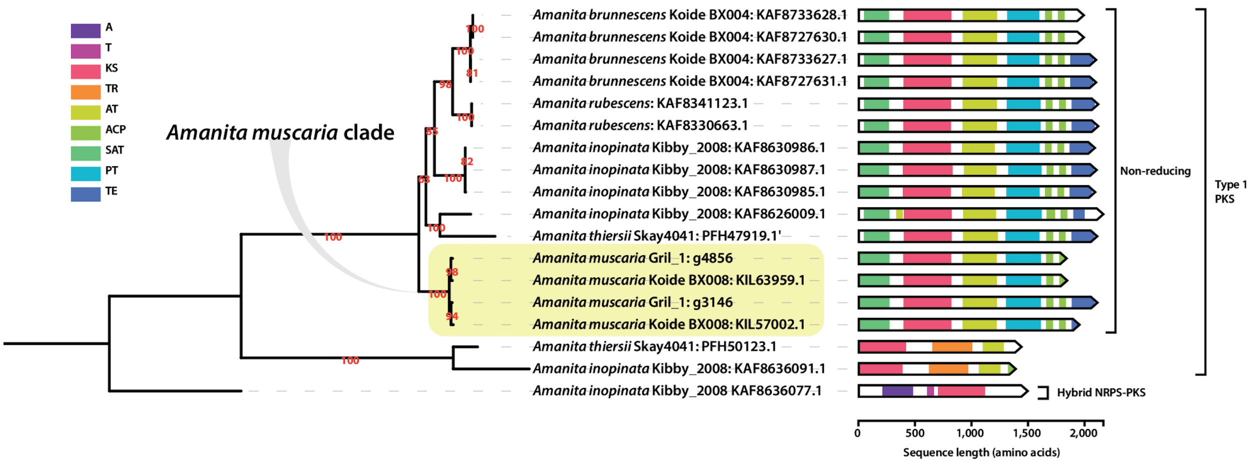

Fig. S9 Comparison of polyketide synthases found in Amanita genomes. A phylogeny of synthase sequences (left). The clade of sequences found in *Amanita muscaria* genomes is highlighted in yellow. Bootstrap support values are indicated in red. The tree is rooted at a Hybrid NRPS-PKS gene from *Amanita inopinata*. The domain structures of corresponding protein sequences are presented on the right with the following domains marked: adenylation (A), thiolation (T), keto synthase (KS), thioester reductase (TR), acetyltransferase (AT), acyl-carrier protein (ACP), starter unit acyltransferase (SAT), product template (PT), thioesterase (TE). A larger analysis of across Agaricomycetes is presented in Figure 4.

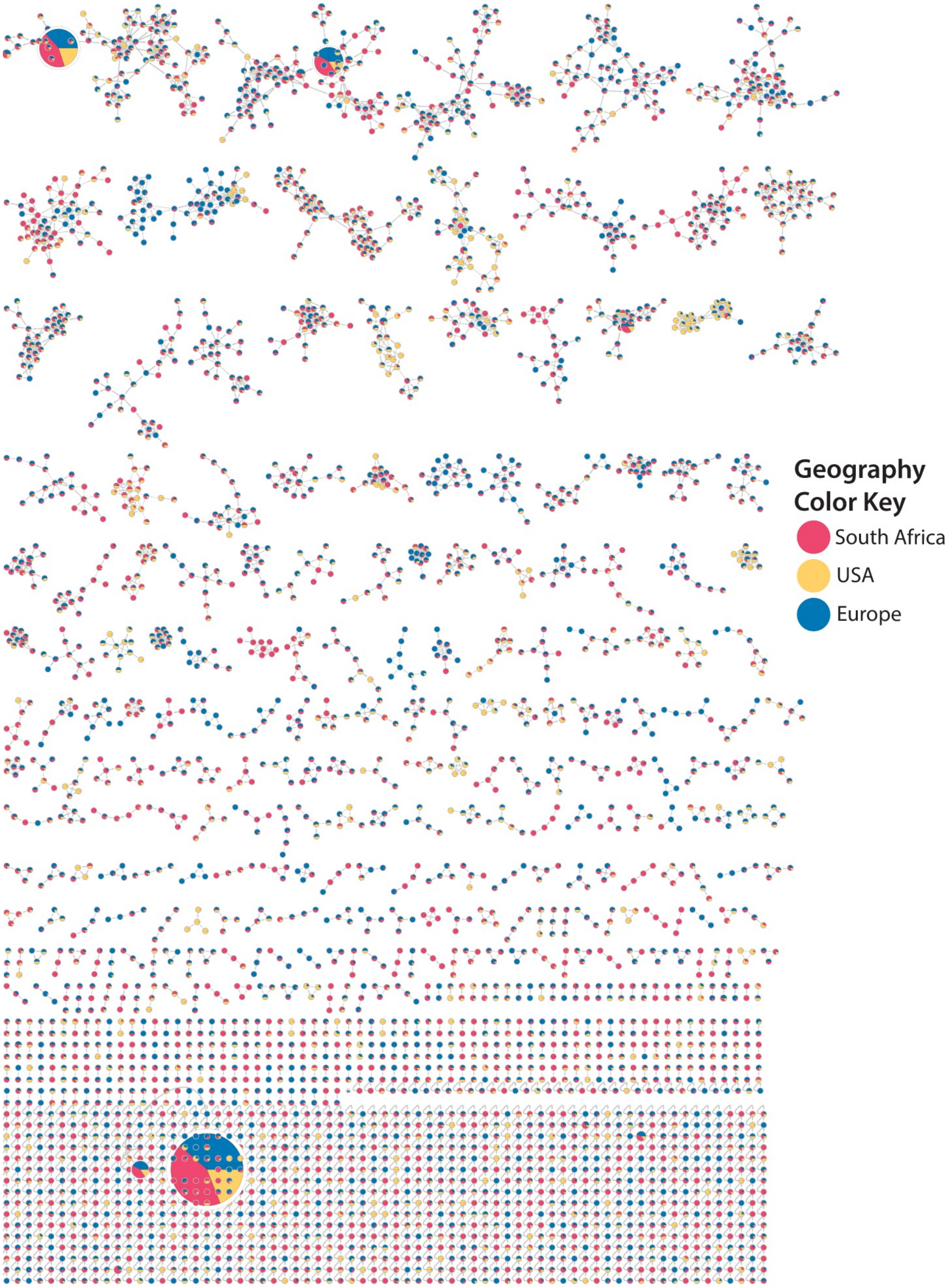
Geography Color Key
South Africa
USA
Europe

Fig. S10 Full GNPS output plot of molecular families, including singletons. Node size reflects the total precursor intensity. Each individual node contains a pie chart showing which regions that unique molecular feature was detected in.

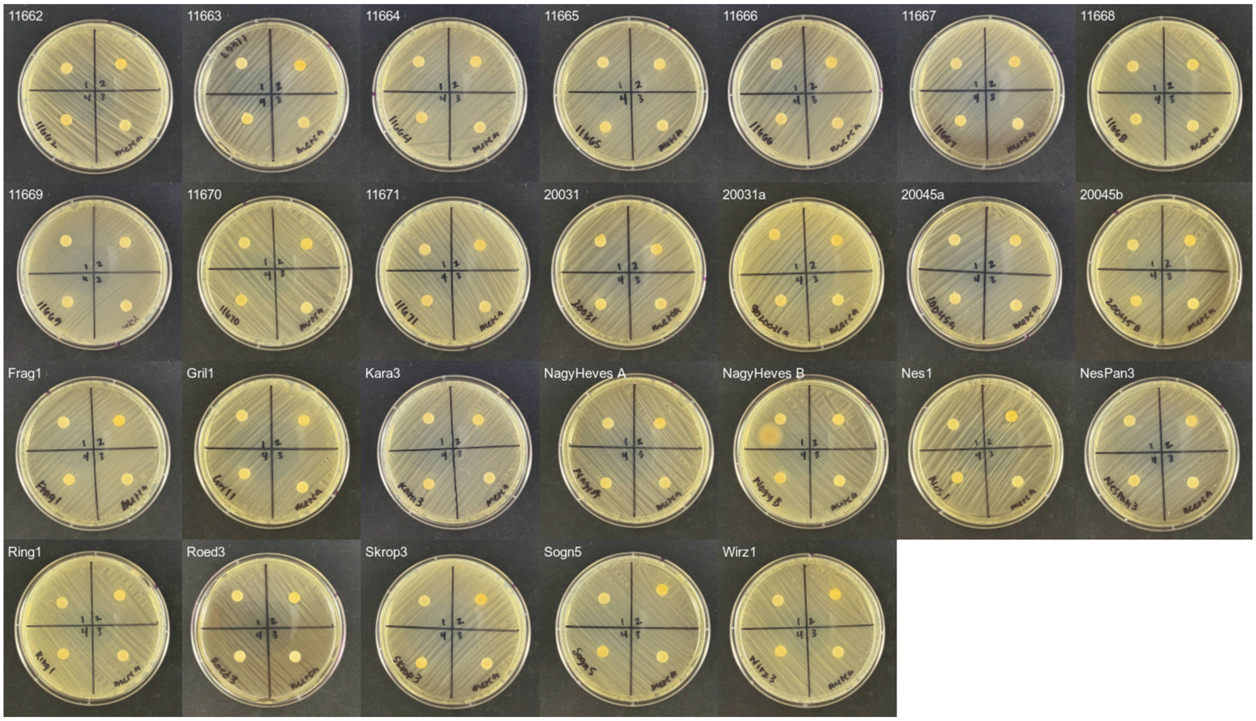

Fig. S11 Bioassays of metabolite extracts against Methicillin-resistant *Staphylococcus aureus.* Each plate represents assays of MRSA against one crude extract. The sample ID that the crude extract came from is indicated in white in the top left of each image. The numbers on each sector correspond to the treatment group as follows: 1) Paper blank, 2) 20 µL of 2.5 mg/mL crude extract, 3) MeOH blank, and 4) 20 µL of 5 mg/mL crude extract.

New Phytologist

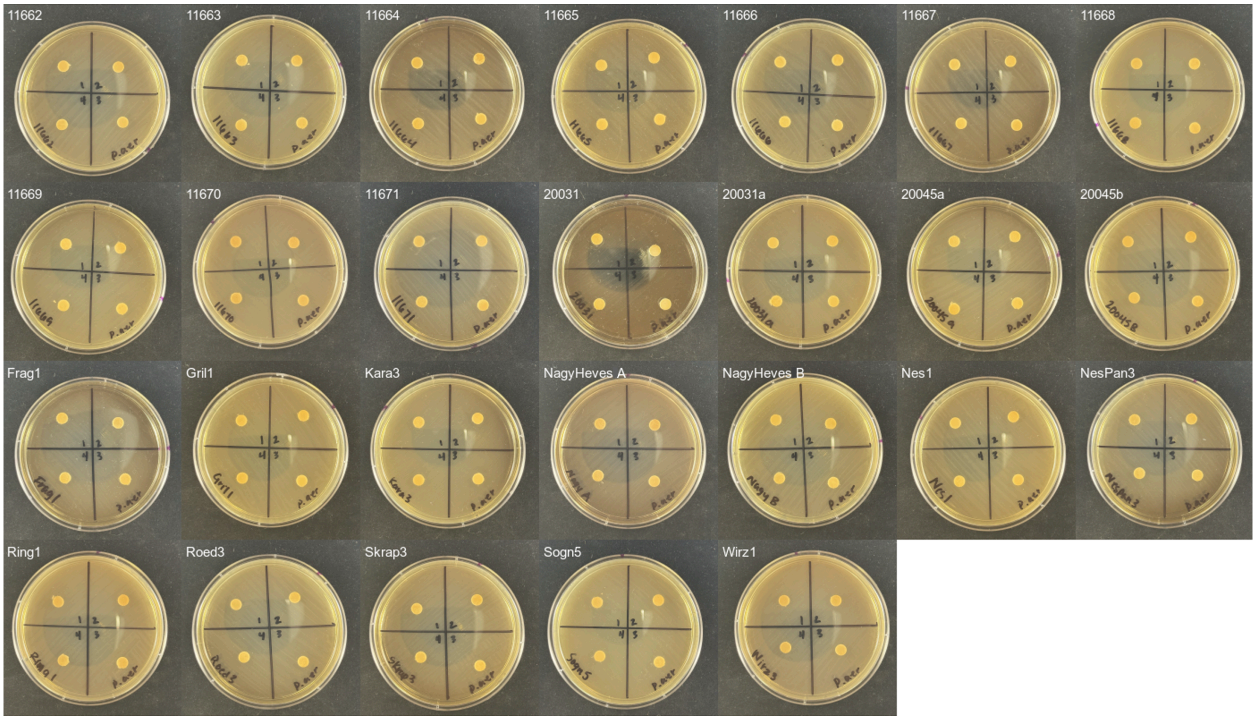

Fig. S12 Bioassays of metabolite extracts against *Pseudomonas aeruginosa.*
Each plate represents assays of *P. aeruginosa* against one crude extract. The sample ID that the crude extract came from is indicated in white in the top left of each image. The numbers on each sector correspond to the treatment group as follows: 1) Paper blank, 2) 20 µL of 2.5 mg/mL crude extract, 3) MeOH blank, and 4) 20 µL of 5 mg/mL crude extract.

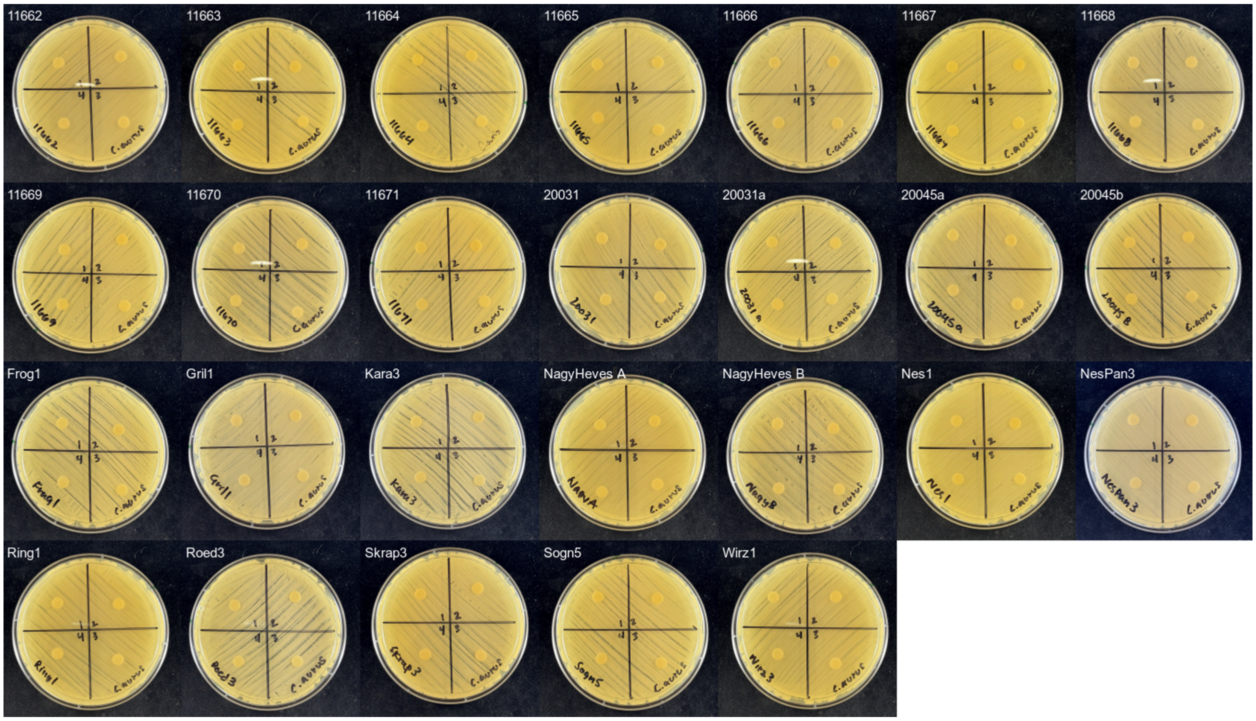

Fig. S13 Bioassays of metabolite extracts against *Candida auris*.
Each plate represents assays of *C. auris* against one crude extract. The sample ID that the crude extract came from is indicated in white in the top left of each image. The numbers on each sector correspond to the treatment group as follows: 1) Paper blank, 2) 20 µL of 2.5 mg/mL crude extract, 3) MeOH blank, and 4) 20 µL of 5 mg/mL crude extract.

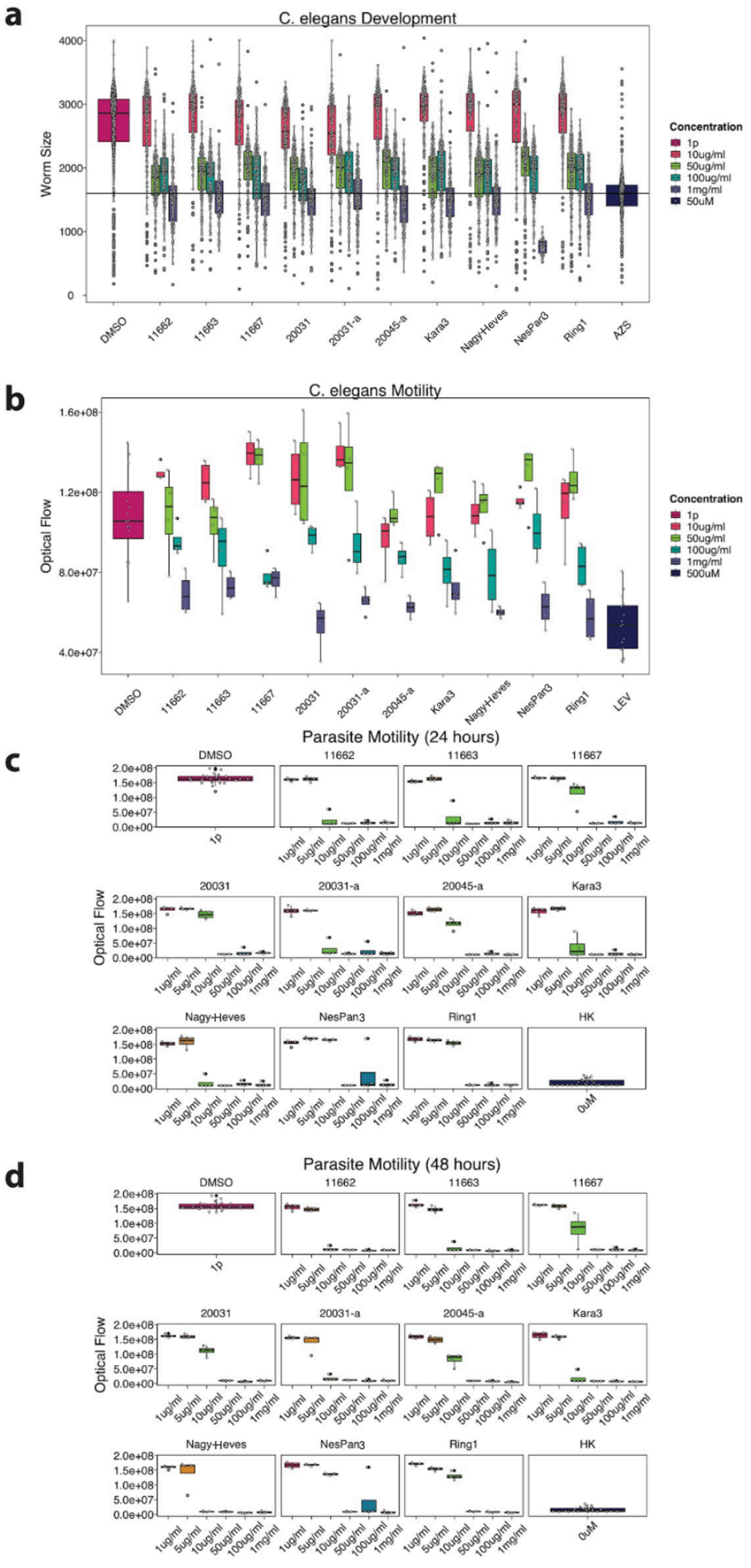
a
C. elegans Development
Worm Size
4000
3000
2000
1000
0
DMSO
11662
11663
11667
20031
20031-a
20045-a
Kara3
Nagy-Heves
NesPan3
Ring1
AZS
Concentration
1p
10ug/ml
50ug/ml
100ug/ml
1mg/ml
50uM
b
C. elegans Motility
Optical Flow
1.6e+08
1.2e+08
8.0e+07
4.0e+07
LEV
500uM
c
Parasite Motility (24 hours)
HK
1ug/ml
5ug/ml
10ug/ml
50ug/ml
100ug/ml
1mg/ml
0uM
2.0e+08
1.5e+08
1.0e+08
5.0e+07
0.0e+00
d
Parasite Motility (48 hours)

New Phytologist

Figure S14 Nematode bioassay results after treatment with several concentrations of extracts. a) *C. elegans* size 48 hours after treatment as L1s shows developmental effects compared to negative control, DMSO, and positive control, Albendazole Sulfoxide (AZS). b) Motility of adult *C. elegans* 20 mins after treatment shows putative neuromuscular effects compared to negative control, DMSO, and positive control, levamisole. c) Motility of *Brugia* microfilariae 24 hours after treatment compared to negative control, DMSO, and positive control, heat killed (HK) microfilariae. d) *Brugia* microfilariae motility 48 hours after treatment. e) *Brugia* microfilariae fluorescence in response to Celltox reagent 48 hours after extract treatment differentiates between paralyzed (non-motile, living) and dead microfilariae.

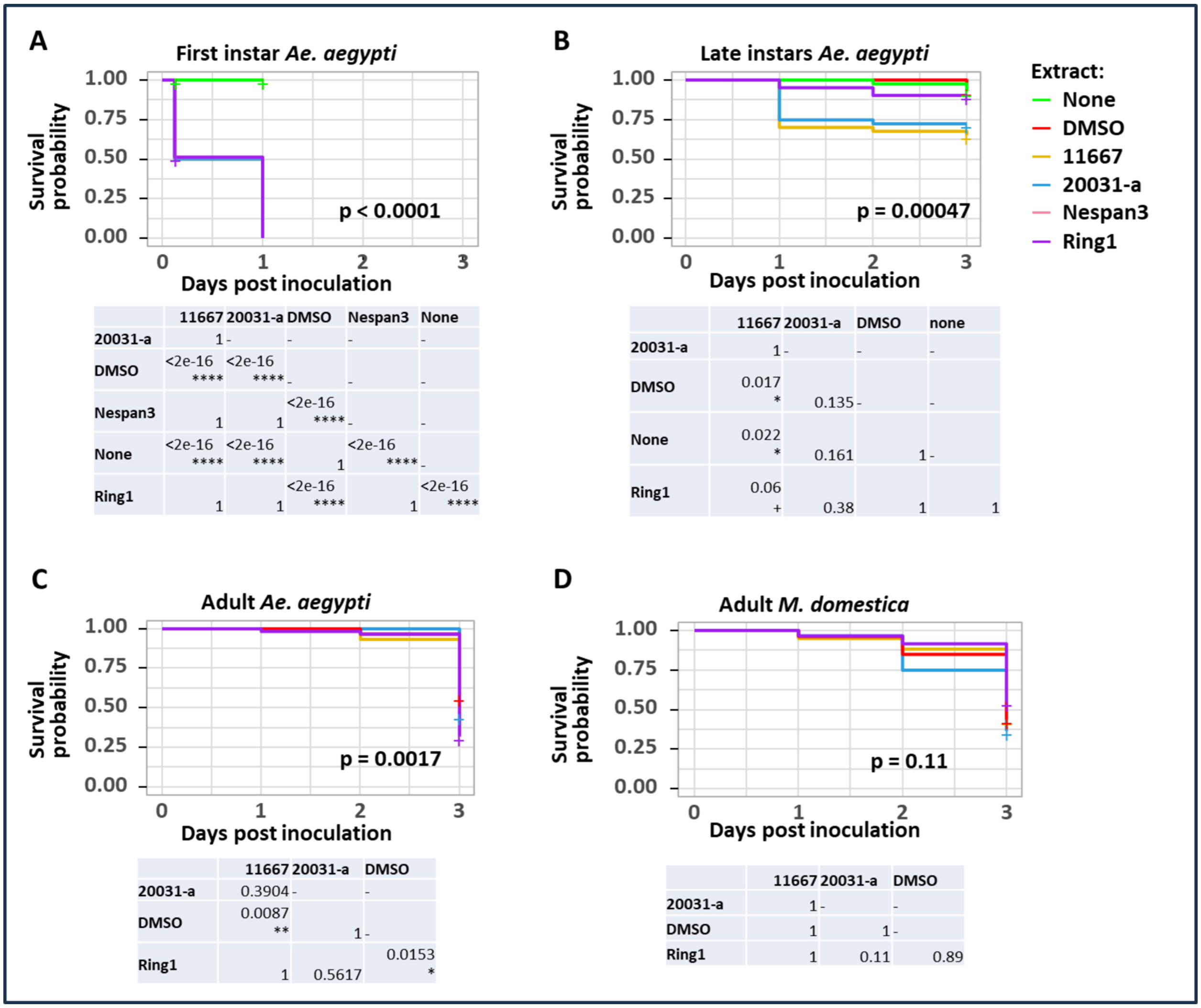

A

	11667	20031-a	DMSO	Nespan3	None
20031-a	1	-	-	-	-
DMSO	<2e-16 ****	<2e-16 ****	-	-	-
Nespan3	1	1	<2e-16 ****	-	-
None	<2e-16 ****	<2e-16 ****	1	<2e-16 ****	-
Ring1	1	1	<2e-16 ****	1	<2e-16 ****

B

	11667	20031-a	DMSO	none
20031-a	1	-	-	-
DMSO	0.017 *	0.135	-	-
None	0.022 *	0.161	1	-
Ring1	0.06 +	0.38	1	1

C

	11667	20031-a	DMSO
20031-a	0.3904	-	-
DMSO	0.0087 **	1	-
Ring1	1	0.5617	0.0153 *

D

	11667	20031-a	DMSO
20031-a	1	-	-
DMSO	1	1	-
Ring1	1	0.11	0.89

Fig. S15 Viability assays in *Ae. aegypti* and *M. domestica*. A-D: (Top) Kaplan-Meier survival curves for different life stages of *Ae. aegypti* and adult *M. domestica*; (Bottom) Bonferroni-corrected log-rank *p*-values of pairwise comparisons of sample survival across treatments. The treatments are extracts from South African *Amanita* (11667), USA (20031-a), the *A. pantherina* from Europe (Nes_pan3), and *A. muscaria* from Europe (Ring_1). None and DMSO are the controls. Significance levels: 0 '****' 1e-04 '***' 0.001 '**' 0.01 '*' 0.05 '+' 0.1 ' '.

New Phytologist

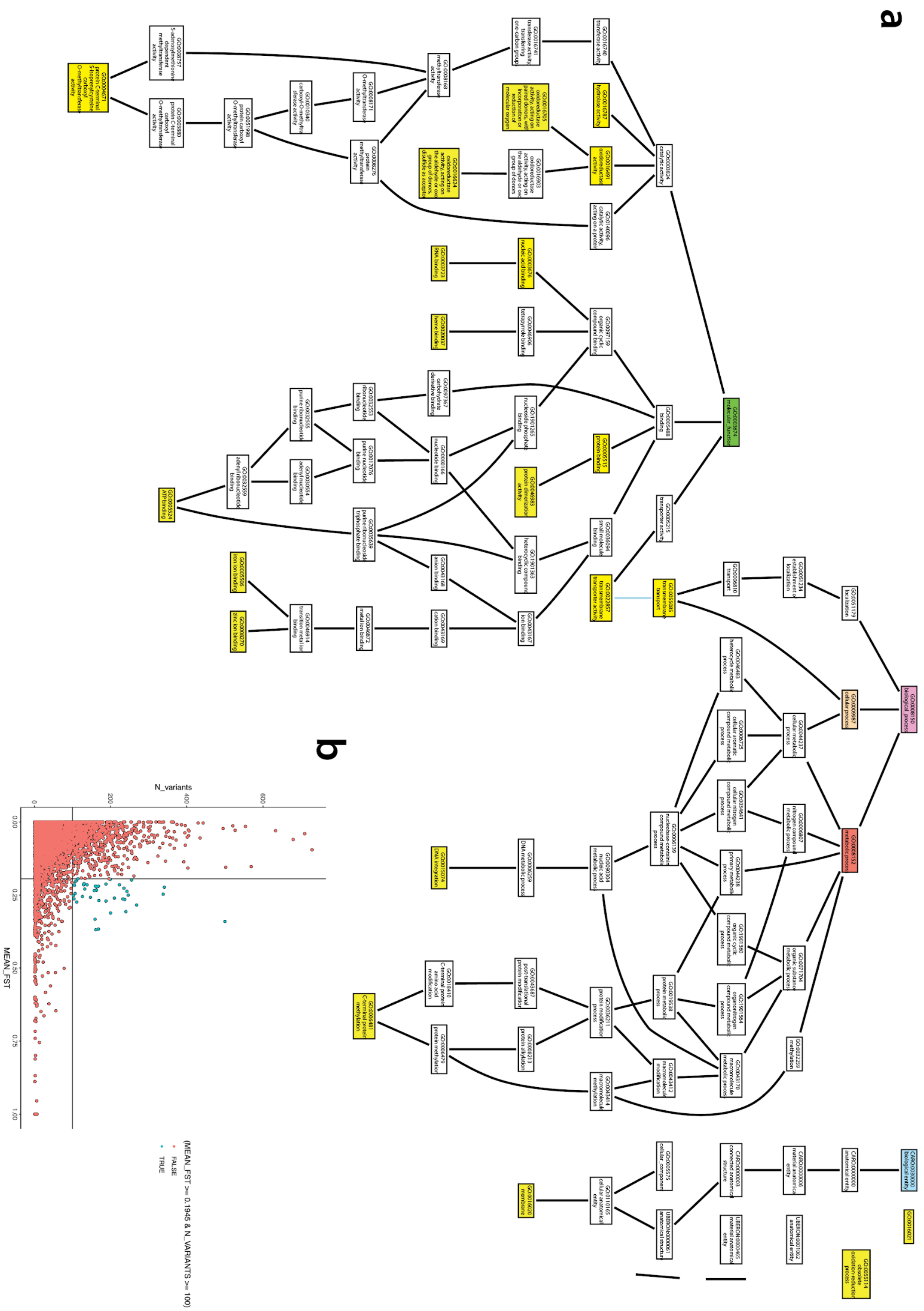

New Phytologist

Figure S16. a) GO-Term map corresponding to genes that overlapped with 5 kb sliding windows containing at least 100 SNPs and where estimates of Fst corresponded to the right 5% tail of estimates.The yellow boxes indicate GO-terms specifically enriched. The white are connecting terms between all of the yellow boxes. The major high level functions such as “metabolic process”, “biological process”, “cellular process” and “biological entity” were given other colors at random to help them stand out. b) A scatterplot showing the distribution of Fst estimates in 5 kb sliding windows. The points corresponding to the regions selected for this GO-Term analysis are indicated in blue.

New Phytologist

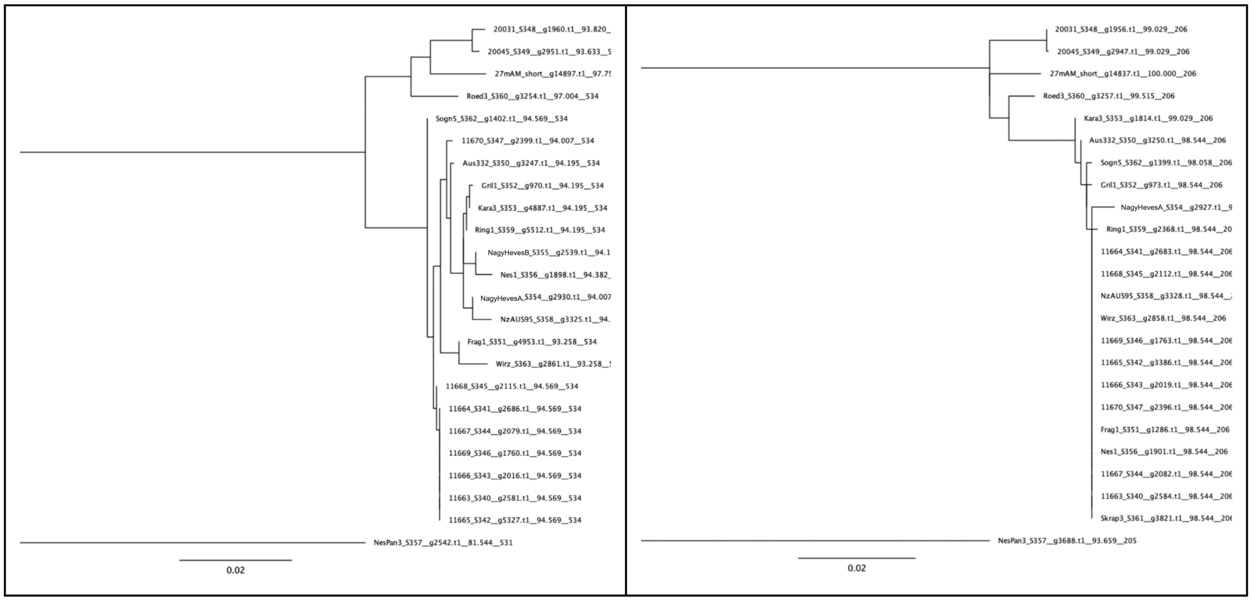

Figure S17: Phylogenetic trees constructed using a codon-aware alignment of the iboF (left) and iboH (right) genes. Tests of relaxed selection conducted comparing the European and South African clades in Phylogenetic Analysis by Maximum Likelihood (PAML) found no significant difference between groups across different models ($P > 0.21$ and $P > 0.163$ for each gene respectively). Similar tests implemented using the RELAX model on Datamonkey webserver yielded similar results ($P > 0.292$ and $P > 0.551$ respectively).

References

Airs PM, Kudrna KE, Bartholomay LC. **2019**. Impact of sugar composition on meal distribution, longevity, and insecticide toxicity in Aedes aegypti. *Acta Tropica* **191**: 221–227.

Capella-Gutiérrez S, Silla-Martínez JM, Gabaldón T. **2009**. trimAl: A tool for automated alignment trimming in large-scale phylogenetic analyses. *Bioinformatics* **25**: 1972–1973.

Coon KL, Brown MR, Strand MR. **2016**. Mosquitoes host communities of bacteria that are essential for development but vary greatly between local habitats. *Molecular Ecology* **25**: 5806–5826.

Drott MT, Park SC, Wang Y, Harrow L, Keller NP, Pringle A. **2023**. Pangenomics of the death cap mushroom Amanita phalloides, and of Agaricales, reveals dynamic evolution of toxin genes in an invasive range. *The ISME Journal*: 1–11.

Huson DH. **1998**. SplitsTree: analyzing and visualizing evolutionary data. *Bioinformatics* **14**: 68–73.

Huson DH, Bryant D. **2006**. Application of Phylogenetic Networks in Evolutionary Studies. *Molecular Biology and Evolution* **23**: 254–267.

Katoh K, Misawa K, Kuma K, Miyata T. **2002**. MAFFT: a novel method for rapid multiple sequence alignment based on fast Fourier transform. *Nucleic Acids Research* **30**: 3059–3066.

Katoh K, Standley DM. **2013**. MAFFT multiple sequence alignment software version 7: Improvements in performance and usability. *Molecular Biology and Evolution* **30**: 772–780.

Kumar S, Stecher G, Li M, Knyaz C, Tamura K. **2018**. MEGA X: Molecular evolutionary genetics analysis across computing platforms. *Molecular Biology and Evolution* **35**: 1547–1549.

Michalski ML, Griffiths KG, Williams SA, Kaplan RM, Moorhead AR. **2011**. The NIH-NIAID Filariasis Research Reagent Resource Center. *PLoS Neglected Tropical Diseases* **5**: e1261.

Minh BQ, Schmidt HA, Chernomor O, Schrempf D, Woodhams MD, Haeseler A von, Lanfear R. **2020**. IQ-TREE 2: New Models and Efficient Methods for Phylogenetic Inference in the Genomic Era. *Molecular Biology and Evolution* **37**: 1530–1534.

Ortiz, M E. **2019**. *vcf2phylip v2. 0: convert a VCF matrix into several matrix formats for phylogenetic analysis*.

Stamatakis A. **2014**. RAxML version 8: a tool for phylogenetic analysis and post-analysis of large phylogenies. *Bioinformatics* **30**: 1312–1313.

Tillich M, Lehwark P, Pellizzer T, Ulbricht-Jones ES, Fischer A, Bock R, Greiner S. **2017**. GeSeq – versatile and accurate annotation of organelle genomes. *Nucleic Acids Research* **45**: W6–W11.

Towns J, Brown S, Katz DS, Miller MA, Pfeiffer W, Schwartz T. **2011**. The CIPRES science gateway. *Proceedings of the 2011 TeraGrid Conference: Extreme Digital Discovery*: 1–8.

Wheeler NJ, Gallo KJ, Rehborg EJG, Ryan KT, Chan JD, Zamanian M. **2022a**. wrmXpress: A modular package for high-throughput image analysis of parasitic and free-living worms. *PLOS Neglected Tropical Diseases* **16**: e0010937.

Wheeler NJ, Ryan KT, Gallo KJ, Henthorn CR, Ericksen SS, Chan JD, Zamanian M. **2023**. Multivariate chemogenomic screening prioritizes new macrofilaricidal leads. *Communications Biology* **6**: 44.

Wheeler NJ, Zamanian M, Ryan KT, Gallo KJ. **2022b**. Bivariate, high-content screening of *Brugia malayi* microfilariae.
